# Continuous Inflammatory Stimulation Leads via Metabolic Plasticity to a Prometastatic Phenotype in Triple-Negative Breast Cancer Cells

**DOI:** 10.3390/cells10061356

**Published:** 2021-05-31

**Authors:** Dina Morein, Linor Rubinstein-Achiasaf, Hadar Brayer, Orly Dorot, Edward Pichinuk, Hagar Ben-Yaakov, Tsipi Meshel, Metsada Pasmanik-Chor, Adit Ben-Baruch

**Affiliations:** 1 Shmunis School of Biomedicine and Cancer Research, George S. Wise Faculty of Life Sciences, Tel Aviv University, Tel Aviv 6997801, Israel; Dinamorein1@gmail.com (D.M.); Linoru@gmail.com (L.R.-A.); hadarbrayer@gmail.com (H.B.); etzion.hagar1@gmail.com (H.B.-Y.); tsipi.meshel@gmail.com (T.M.); 2 Blavatnik Center for Drug Discovery, Tel Aviv University, Tel Aviv 6997801, Israel; orly.dorot@gmail.com (O.D.); eddypichinuk@gmail.com (E.P.); 3 Bioinformatics Unit, George S. Wise Faculty of Life Sciences, Tel Aviv University, Tel Aviv 6997801, Israel; metsada@tauex.tau.ac.il

**Keywords:** glycolysis, inflammation, interleukin 1β, leukocyte migration, metabolism, oxidative phosphorylation, triple-negative breast cancer, tumor necrosis factor α

## Abstract

Chronic inflammation promotes cancer progression by affecting the tumor cells and their microenvironment. Here, we demonstrate that a continuous stimulation (~6 weeks) of triple-negative breast tumor cells (TNBC) by the proinflammatory cytokines tumor necrosis factor α (TNFα) + interleukin 1β (IL-1β) changed the expression of hundreds of genes, skewing the cells towards a proinflammatory phenotype. While not affecting stemness, the continuous TNFα + IL-1β stimulation has increased tumor cell dispersion and has induced a hybrid metabolic phenotype in TNBC cells; this phenotype was indicated by a transcription-independent elevation in glycolytic activity and by increased mitochondrial respiratory potential (OXPHOS) of TNBC cells, accompanied by elevated transcription of mitochondria-encoded OXPHOS genes and of active mitochondria area. The continuous TNFα + IL-1β stimulation has promoted in a glycolysis-dependent manner the activation of p65 (NF-κB), and the transcription and protein expression of the prometastatic and proinflammatory mediators sICAM-1, CCL2, CXCL8 and CXCL1. Moreover, when TNBC cells were stimulated continuously by TNFα + IL-1β in the presence of a glycolysis inhibitor, their conditioned media had reduced ability to recruit monocytes and neutrophils in vivo. Such inflammation-induced metabolic plasticity, which promotes prometastatic cascades in TNBC, may have important clinical implications in treatment of TNBC patients.

## 1. Introduction

Cancer-related inflammation has been identified as a key process controlling tumor progression. Whereas typical Th1-related/inflammatory mediators such as interferon γ can have essential roles in potentiating acquired antitumor immune activities at initial stages of tumor development, inflammation often turns into a chronic condition that promotes tumor growth and metastasis [1,2,3,4]. Many malignancies are enriched with inflammatory elements that include myeloid cells with inflammatory or immune-suppressive functions, as well as proinflammatory cytokines and inflammatory chemokines. Due to the promalignancy activities of these different proinflammatory constituents, the establishment of primary tumors and the metastatic spread are potentiated, leading eventually to poor patient survival [3,4,5].

In breast cancer, inflammatory cells and inflammatory soluble mediators have been clearly connected with a highly metastatic phenotype. For example, potent proinflammatory cytokines such as tumor necrosis factor α (TNFα) and interleukin 1β (IL-1β) have been connected with increased aggressiveness in breast cancer [6,7,8,9,10,11]: their expression levels were significantly associated with tumor progression and poor clinical outcome of breast cancer patients [12,13,14,15,16,17] and they were both found to express a large variety of tumor-promoting activities in culture systems and in animal model systems [6,7,8,9,10,11]. 

A closer look at the expression of TNFα and IL-1β in biopsies of breast cancer patients revealed that in many breast cancer patient tumors the two proinflammatory cytokines were expressed in a chronic manner, starting in low tumor grades and from the stage of ductal carcinoma in situ and on, eventually being expressed in tumors of ~90% of patients with advanced disease [13,14,18]. Moreover, in most patient tumors TNFα and IL-1β were concomitantly expressed at the tumor site and in patient materials [13,14,18]. 

In view of these observations, in this study we determined the impact of continuous proinflammatory stimulation by both TNFα + IL-1β together on the malignancy phenotype of breast cancer cells. Particularly, we focused on triple-negative breast cancer (TNBC), which is the most aggressive subtype of breast cancer [19,20,21], requiring much improved understanding of basic processes taking place during disease progression. So far, TNBC cells were found to respond to short-term TNFα or IL-1β stimulation (up to 72 h) by increased proliferation and migration, as well as the release of angiogenic factors, MMP-9, osteopontin and proinflammatory chemokines [22,23,24,25,26,27,28]. However, the impacts of consistent proinflammatory stimulation by TNFα + IL-1β—reflecting chronic inflammatory processes that take place at the tumor site—were not investigated in TNBC.

Thus, in this study we exposed TNBC cells to a persistent stimulation by TNFα + IL-1β together and determined the effects of this stimulatory setup on tumor cell phenotypes and activities. The current study provides novel findings, indicating that continuous growth of TNBC cells with the two cytokines has led to potentiation of metabolic activities in the tumor cells. Particularly, following persistent TNFα + IL-1β stimulation, the ability of TNBC cells to perform glycolysis and oxidative phosphorylation (OXPHOS) was significantly increased. Moreover, glycolysis has increased the activation of NF-κB, and the transcription and protein expression of proinflammatory mediators that are typically connected to elevated aggressiveness of tumor cells, e.g., by their ability to recruit deleterious myeloid cell types to tumors. Accordingly, when TNBC cells were continuously stimulated by TNFα + IL-1β, their ability to secrete factors that induce the recruitment of monocytes and neutrophils to matrigel plugs in vivo was reduced by treating the tumor cells with a glycolysis inhibitor.

These observations suggest that chronic presence of proinflammatory cytokines such as TNFα + IL-1β at the tumor microenvironment (TME) drives TNBC cells into a prometastatic phenotype, in a manner that has not been reported previously; such a persistent proinflammatory stimulation can modify through metabolic upregulation the expression of inflammatory mediators that indirectly, through the recruitment of deleterious monocytes and neutrophils, may increase the prometastatic phenotype of TNBC cells. The findings of this current study—demonstrating that a persistent inflammatory stimulation of TNBC cells leads to a hybrid metabolic state with increased glycolysis as well as of OXPHOS activities—emphasize the need to perform extensive research in this direction in the future, and suggest that combination therapies that target tumor cell metabolism and inflammation may apply well in the treatment of TNBC patients.

## 2. Materials and Methods

### 2.1. Cell Cultures and Cytokine Stimulation

Human TNBC BT-549 cells and MDA-MB-231 cells (both from ATCC) were grown in DMEM (4.5 g/L glucose) and RPMI-1640 (2 g/L glucose) media, respectively. To generate “complete media”, the media were supplemented with 10% fetal bovine serum (FBS), 2% L-glutamine and 1% penicillin-streptomycin solution (all from Biological Industries, Beit Ha’emek, Israel). 

TNFα and IL-1β concentrations were selected based on doses used in other studies and on titration analyses that were previously performed in the lab. Recombinant human (rh) TNFα was used at 10 ng/mL (#300-01A, PeproTech, Rocky Hill, NJ, USA) and rhIL-1β was used at 0.4ng/mL (#200-01B, PeproTech), at two different regimens: (1) Continuous stimulation: TNBC cells were grown for ~6 weeks in the presence of the two cytokines together (replaced twice a week). (2) Short stimulation: TNBC cells were stimulated by TNFα + IL-1β (similar concentrations as in continuous stimulation) for 48 h only. In both stimulatory conditions (continuous and short) control cells were grown with the vehicle of the cytokines.

The process of continuous stimulation was done three times for each cell type. Each process of continuous stimulation was considered a separate “set”, and the cells of each set were frozen at the end of the continuous stimulation process. To be used in experiments, the cells were thawed and brought back to culture in the presence of cytokines or vehicle (as appropriate), in parallel. In each type of experiment, similar findings were obtained with at least two different sets of cells, in BT-549 and MDA-MB-231 cells alike. 

Monocytic THP-1 cells were grown in suspension, in RPMI-1640 medium supplemented with 20% FBS, 2% L-glutamine, 1% penicillin-streptomycin solution, 1% MEM nonessential amino acids (all from Biological Industries) and 0.05 mM 2-mercapthoethanol (#M6250, Sigma-Aldrich, Saint Louis, MO, USA).

### 2.2. Morphology Analyses and IN Cell Studies

In morphology studies, TNBC cells that have undergone continuous treatment by TNFα + IL-1β/vehicle were photographed using light microscopy. In IN Cell analyses, TNBC cells were cultured for 72 h in complete media containing TNFα + IL-1β/vehicle under automated high-throughput screening conditions (Freedom EVO 200 robot, Tecan Group Ltd., Männedorf, Switzerland). The cells were stained and imaged by the IN Cell Analyzer 2200 automated microscope (GE Healthcare, Chicago, IL, USA) at 20× magnification. Cell and nucleus areas were determined by cytoplasmic and nuclear staining using calcein (#C3100MP, Thermo Fisher Scientific, Waltham, MA, USA) and Hoechst (#H1399, Thermo Fisher Scientific), respectively. Cellular organelles were stained with MitoTracker-Deep Red (overall mitochondria; #M22426, Thermo Fisher Scientific), tetramethylrhodamine (TMRE; active mitochondria; #T669, Thermo Fisher Scientific), lysotracker (lysosome; #L12492, Thermo Fisher Scientific) or ER-hunt (endoplasmic reticulum; #7305, Setarech biotech, Eugene, OR, USA). Following image acquisition, high-content image stacks were analyzed using the IN Cell developer Toolbox 1.9.3 (GE Healthcare) producing an output based on comparative fluorescence intensity.

### 2.3. Transcriptome Analyses

To determine transcriptome alterations following continuous TNFα + IL-1β stimulation in TNBC cells, genome-wide expression analysis was performed on BT-549 and MDA-MB-231 cells in RNAseq experiments. The analysis of each cell type was conducted on three independent biological repeats of treatment with cytokines/vehicle.

In BT-549 cells, RNA libraries were prepared using NEBNext^®^ Poly(A) mRNA Magnetic Isolation Module (#E7490, New England Biolabs, Ipswich, MA, USA), then submitted to analysis using NextSeq 500/550 v2.5 high output kit (#20024906; New England Biolabs) for RNAseq at the Genomics Unit of Tel Aviv University. Samples were sequenced on four lanes of Illumina Nextseq500. The output was ~20 million reads per sample. Trimming was performed on low-quality reads. In MDA-MB-231 cells, total RNA was isolated using miRNeasy Mini Kit (#217004, QIAGEN, Hilden, Germany), then submitted to RNAseq analysis at the Israel National Center for Personalized Medicine (INCPM, Weizmann Institute, Rehovot, Israel). Libraries were prepared at the INCPM from the RNA samples using in-house protocol. Samples were sequenced on eight lanes of Illumina HiSeq-2500 machine, using the Single-Read 60 protocol. 

The processed reads in each cell type were mapped to human genome, GRCh38, using STAR v2.6.1d [29] and then the number of reads that were mapped to each gene was calculated using Partek E/M algorithm. Data of transcriptome analyses of BT-549 and MDA-MB-231 cells were deposited in NCBI’s Gene Expression Omnibus [30] and are accessible through GEO Series accession number GSE169018. Statistical analyses were performed by one-way ANOVA statistical test at cutoff of fold change (FC) ≥ 2 or FC ≤ −2; pFDR was applied on the samples for subsequent analysis, as indicated in Figure legends. Heatmaps were generated using Partek Genomics Suite (Partek Genomics Suite^®^; version 7.19.1125), using hierarchical clustering with Pearson’s correlation distance dissimilarity measures and Ward’s method. For pathway gene enrichment analysis, 985 differentially expressed genes in BT-549 cells and 779 differentially expressed genes in MDA-MB-231 cells, were analyzed in the Ingenuity software. Protein–protein association networks were analyzed by STRING v11 [31].

### 2.4. Cell Counts and XTT Assays

TNBC cells were continuously treated by TNFα + IL-1β/vehicle. To determine cell proliferation, cytokine- and vehicle-treated cells were plated at similar numbers, and cells were counted by trypan blue-exclusion assay after 72 h in several replicates for each treatment. XTT assays were performed (#20-30-1000, Biological Industries) on cells that were seeded 72 h earlier in the presence of TNFα + IL-1β/vehicle. Doxorubicin (5 μM; Teva Pharmaceutical, Netanya, Israel; kindly provided by Prof. Peer, Tel Aviv University) and/or paclitaxel (350 nM; #T7402, Sigma-Aldrich) were added to the final 24 h of the incubation, in the presence of the cytokines or vehicle. The control for chemotherapy treatment was the solubilizer of the drugs.

### 2.5. FACS Analyses 

TNBC cells that were continuously treated by TNFα + IL-1β/vehicle have been analyzed for the expression of the following molecules: (1) CD44 and CD24 (cancer stem cells, CSCs)—staining with Alexa 488-tagged rat antibodies to human CD44 (#103016, Biolegend, San Diego, CA, USA) and with PE-tagged mouse antibodies to human CD24 (#560991, BD Biosciences, San Jose, CA, USA); (2) surface E-cadherin—staining with APC-tagged mouse antibodies to human E-cadherin (#324107, Biolegend); (3) vimentin—analysis of cells fixed and permeabilized with 100% methanol (10 min, −20 °C), stained by mouse antibodies to human vimentin (#sc-6260, Santa Cruz Biotechnology, Dallas, TX, USA) followed by Alexa 647-tagged goat antimouse antibody (#115-606-146, Jackson ImmunoResearch Laboratories, West Grove, PA, USA). In all cases, baseline staining was determined by relevant isotype-control antibodies. Staining was assessed using Stratedigm S1000EXi flow cytometry analyzer (Stratedigm, San Jose, CA, USA) and data analysis was performed using the Flowjo v10 software (BD Biosciences).

### 2.6. Spheroid Formation

mCherry-expressing TNBC cells that were continuously treated by TNFα + IL-1β/vehicle were cultured overnight in plates coated by poly-HEMA (#P3932, Sigma-Aldrich) in DMEM/F12 medium containing 2% B-27 supplement, 0.02% basic fibroblast growth factor, 0.2% epidermal growth factor and 0.05% insulin to enable spheroid formation. The spheroids were maintained in a 37 °C incubator with 5% CO_2_. Kinetics of spheroid formation were tracked using fluorescence microscopy, and representative images were taken after 3 days for BT-549 cells and after 7 days for MDA-MB-231 cells, respectively, at 4× magnification. Based on fluorescent signals, the size of spheroids was quantified by the ImageJ program in 10 fields for each treatment, in each experiment. 

### 2.7. Seahorse XF Analysis

TNBC cells were continuously treated by TNFα + IL-1β/vehicle. Oxidative phosphorylation and glycolytic rates of the cells were assessed using a Seahorse XFe96 Analyzer (Agilent Technologies Inc., Santa Clara, CA, USA). To this end, TNBC cells were cultured in XF-96-well cell culture plates (Agilent). After 24 h the media were replaced by fresh complete media with TNFα + IL-1β/vehicle for 48 h. For the mito stress test, oxygen consumption rate (OCR) measurements were taken at basal rate followed by injections of oligomycin (1 μM; #O4876, Sigma-Aldrich), carbonyl cyanide 4-(trifluoromethoxy)phenylhydrazone (FCCP; 1 μM; #C2920, Sigma-Aldrich) and a mixture of rotenone (Rot; 0.5 μM; #R8875, Sigma-Aldrich) + antimycin A (AA; 0.5 μM; #A8674, Sigma-Aldrich). For the glycolytic rate assay, extracellular acidification rate (ECAR) measurements were taken at basal rate followed by injections of mixture of Rot (0.5 μM; #ab143145, Abcam, Cambridge, MA, USA) +AA (as above), and then 2-Deoxy-D-Glucose (2-DG; 50 mM; #ab142242, Abcam).

Following analysis, the cells were fixed with 4% paraformaldehyde and stained with Hoechst (#B2261, Sigma-Aldrich). Each well was photographed at different regions and metabolic signals were normalized to cell numbers.

### 2.8. Determination of Lactate and ATP Levels

TNBC cells that were continuously treated by TNFα + IL-1β/vehicle were cultured in complete media supplemented with TNFα + IL-1β/vehicle. Twenty-four hours prior to the assay, media were replaced by fresh media containing 0.5% FBS (for lactate assays) or 10% FBS (for ATP assays), in the presence of TNFα + IL-1β/vehicle. Extracellular lactate levels in the CM were determined using L-Lactate Assay Kit (#ab65331, Abcam) according to manufacturer’s instructions. The results were normalized to cell counts at the time of CM collection. ATP levels were determined in cell lysates using ATP Assay Kit (#ab83355, Abcam) according to manufacturer’s instructions. Prior to analysis in the kits, cell CM and lysates were deproteinized as recommended, using Amicon Ultra-0.5 Centrifugal Filter Units (#UFC501024, Millipore, Temecula, CA, USA).

### 2.9. ELISA Assays

TNBC cells that were continuously treated by TNFα + IL-1β/vehicle were cultured overnight in complete media supplemented with TNFα + IL-1β/vehicle. CCL2, CXCL8 and CXCL1 levels were determined in CM collected after additional 24 h of treatment with TNFα + IL-1β/vehicle, in the presence of metabolic inhibitors and 0.5% FBS-containing media. To determine the levels of soluble intercellular adhesion molecule 1 (sICAM-1), the inhibitors were added 2 h prior the addition of TNFα + IL-1β/vehicle; the inhibitors were then present during the last 24 h of cell culture. After 24 h, the conditioned media (CM) were collected, cleared by centrifugation and ELISA analyses were performed.

The metabolic inhibitors included: (1) the glycolysis inhibitor 2-DG that was used at 50 mM (#ab142242, Abcam). (2) OXPHOS inhibitors: Rot (#ab143145, Abcam)—0.1 μM for MDA-MB-231 cells, 0.05 μM for BT-549 cells + AA (#A8674, Sigma-Aldrich)—0.1 μM for MDA-MB-231 cells, 0.05 μM for BT-549 cells. 

ELISA analyses were performed on cell CM with the following materials (all from PeproTech): for sICAM-1, human ICAM-1 development ELISA kit #900-K464; for CCL2, coating antibodies #500-M71, detection antibodies #500-P34BT and rhCCL2 standard #300-04; for CXCL8, coating antibodies #500-P28, detection antibodies #500-P28BT and rhCXCL8 standard #200-08; and for CXCL1, coating antibodies #500-P92, detection antibodies #500-P92BT and rhCXCL1 standard #300-11. Following the addition of HRP-conjugated Streptavidin (#016-030-084, Jackson Immunoresearch laboratories) and substrate TMB/E solution (#ES001, Millipore), the reaction was stopped by addition of 0.18M H_2_SO_4_. Absorbance was measured at 450 nm.

### 2.10. RNA Extraction and Quantitative RT-PCR

TNBC cells that were continuously treated by TNFα + IL-1β/vehicle were cultured overnight in complete media supplemented with TNFα + IL-1β/vehicle. Then, the media were replaced by fresh DMEM containing 0.5% FBS, the glycolysis inhibitor 2-DG or the OXPHOS inhibitors Rot+AA (concentrations as in ELISA assays), without TNFα + IL-1β/vehicle. Control cells were supplemented with the inhibitor solubilizer. After 2 h, TNFα + IL-1β/vehicle were added to the cells without a wash, for 6 h. RNA was extracted using EZ RNA extraction kit (#20-400-100, Biological Industries). RNA samples were used to synthesize complementary cDNA using qScript^TM^ cDNA synthesis kit (#95047, Quantabio, Beverly, MA, USA). The samples were analyzed using iTaq™ Universal SYBR^®^ Green Supermix (#1725124, BIO-RAD, Hercules, CA, USA) in CFX connect real-time PCR system with primers indicated in Table S1. 

### 2.11. Western Blot Analysis

MDA-MB-231 cells that were continuously treated by TNFα + IL-1β/vehicle were cultured overnight in complete media supplemented with TNFα + IL-1β/vehicle. In specific experiments (as indicated), the media were replaced with fresh DMEM containing 0.5% FBS and were supplemented with OXPHOS inhibitors (Rot+AA) or glycolysis inhibitor (2-DG) (concentrations as in ELISA assays), without TNFα + IL-1β/vehicle. Control cells were supplemented with the inhibitor solubilizer. After 2 h, TNFα + IL-1β/vehicle were added to the cells without a wash, and lysates of cells were made after 15 min or 24 h using RIPA buffer. Total-p65 and phosphorylated p65 (p-p65) were detected using specific antibodies (#8242 and #3033, respectively; Cell Signaling Technology, Danvers, MA, USA). GAPDH was used as loading control (detected by antibodies #ab9485, Abcam). The membranes were reacted with streptavidin-horseradish peroxidase (HRP)-conjugated goat anti-rabbit IgG (#111-035-003, Jackson Immunoresearch laboratories), and subjected to ECL analysis (#WBLUR0500, Merck, Darmstadt, Germany).

### 2.12. Transwell Migration Assay

Migration of TNBC cells: TNBC cells were continuously treated by TNFα + IL-1β/vehicle. Migration assay of TNBC cells towards 10% FBS were for 11 h using transwell inserts with 8 μm pores (#3422, Corning, Corning, NY, USA) that were precoated with fibronectin (20 µg/mL, diluted in serum-free DMEM; #03-090-1; Biological Industries) for 1 h at 37 °C.

Migration of THP-1 cells: MDA-MB-231 cells that were continuously stimulated by TNFα + IL-1β were cultured overnight in complete media supplemented with TNFα + IL-1β. Then, the media were replaced with fresh DMEM containing 0.5% FBS and were supplemented with the glycolysis inhibitor (2-DG) (concentration as in ELISA assays), without TNFα + IL-1β. Control cells were supplemented with the inhibitor solubilizer. After 2 h, TNFα + IL-1β were added without a wash. After 24 h, the media were washed and replaced by fresh TNFα + IL-1β-deprived and inhibitor-free DMEM, which did not contain FBS. After 24 h the CM were collected, filtered through 0.45 μm membrane to discard of cells and concentrated 2×. Then, migration assay of THP-1 cells towards the CM were performed for 75–90 min using transwell inserts with 5 μm pores (#3421, Corning).

In both analyses, the migrated cells were fixed and stained using Hemacolor (#111661, Merck), were photographed and counted at multiple fields.

### 2.13. In Vivo Plug Assay

MDA-MB-231 cells that were continuously stimulated by TNFα + IL-1β were cultured overnight in complete media supplemented with TNFα + IL-1β. Then, the media were replaced with fresh DMEM containing 0.5% FBS and supplemented with glycolysis inhibitor (2-DG) (concentrations as in ELISA assays), without TNFα + IL-1β. Control cells were supplemented with the inhibitor solubilizer. After 2 h, TNFα + IL-1β were added without a wash. After an additional 24 h, the media were washed and replaced by fresh TNFα + IL-1β-deprived and inhibitor-free DMEM, which did not contain FBS. After 48 h the CM were collected, filtered through 0.45 μm membrane to discard of cells and concentrated 2×. The media were mixed with growth factor reduced-matrigel without phenol red (#356231, Corning) in ratio 1:4. 200 μL of CM+matrigel mixture was injected to the mammary fat pad of female Balb/C mice. Procedures involving experimental animals were approved by Tel Aviv University Ethics Committee (Approval no. 04-20-034, dated 18 August 2020), and were performed in compliance with local animal welfare laws, guidelines and policies.

The plugs were removed after 7 days and were solubilized in 1ml HBSS (Biological industries) containing 5 mg/mL collagenase type IV (#C5138; Sigma-Aldrich) and DNase I (#D5025, Sigma-Aldrich) for 1 h at 37 °C. Recovered infiltrating cells were washed three times in PBS and filtered through a 70-μm cell strainer. The recovered cells were counted and incubated for 10 min with TruStain FcX™ (#101320, Biolegend) in order to block non-specific binding to Fc receptors. The cells were then subjected to flow cytometry analysis using the following antibodies: APC-Cy7-tagged antibodies to mouse CD45 (#103116, Biolegend), FITC-tagged antibodies to mouse F4/80 (#123107, Biolegend) and Brilliant Violet-421-tagged antibodies to mouse Ly6G (#127627, Biolegend). Staining was assessed using Stratedigm S1000EXi flow cytometry analyzer (Stratedigm) and data analysis was performed using the Flowjo v10 software (BD Biosciences).

### 2.14. Statistical Analyses

Statistical analyses of transcriptome studies were described in their relevant sections. Statistical analyses of all other assays were performed by two-tailed unpaired Student’s *t*-tests. *p* < 0.05 was considered statistically significant.

## 3. Results

### 3.1. Continuous Stimulation by Proinflammatory Cytokines Induces Morphological Alterations in TNBC Cells

**Figure 1:**
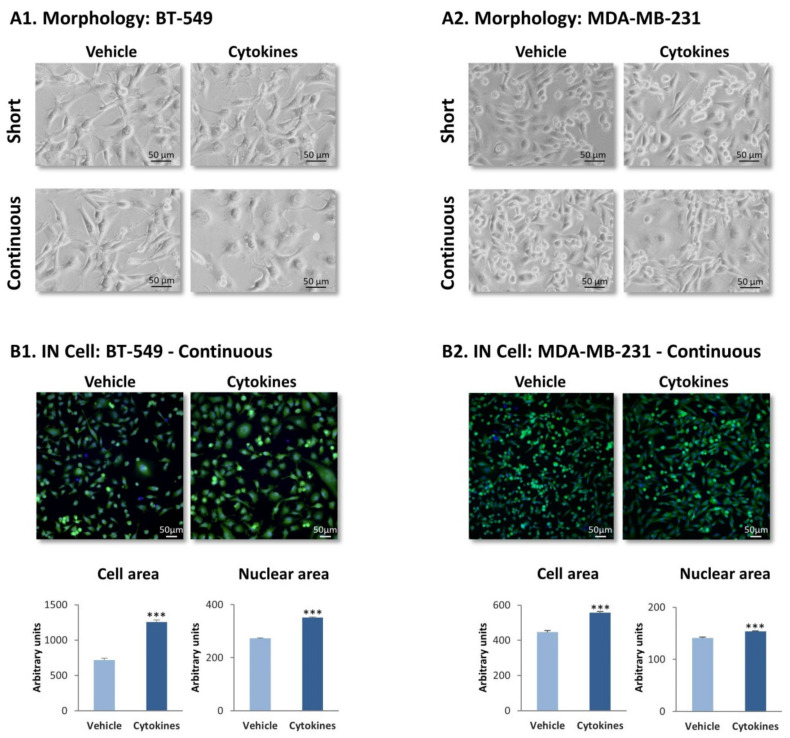
**Continuous TNFα + IL-1β stimulation leads to morphology changes in TNBC cells.** TNFα (10 ng/mL) + IL-1β (0.4 ng/mL) were used to continuously stimulate BT-549 and MDA-MBA-231 cells for ~6 weeks (“continuous” stimulation) or to stimulate the cells for 48 h (“short” stimulation); control cells were treated for the same time periods by the vehicle of the cytokines. Cytokine concentrations were selected based on the considerations described in the materials and methods section. (**A**) Tumor cell morphology determined by light microscopy. (**A1**) BT-549 cells. (**A2**) MDA-MB-231 cells. Phase-contrast images from a representative experiment of *n* > 3 are presented. Bar, 50 μm. (**B**) Determination of cell morphology (images), cell area and nuclear area by the IN Cell technology, using calcein (green) and Hoechst (blue) staining. (**B1**) BT-549 cells. (**B2**) MDA-MB-231 cells. Images of cell morphology are accompanied by quantification of cell characteristics by the IN Cell technology. Bar, 50 μm. The results of a representative experiment of *n* = 3 are presented. *** *p* < 0.001.

To reveal the effects of continuous stimulation by TNFα + IL-1β on TNBC cells we determined the morphology of BT-549 and MDA-MB-231 cells that were stimulated with the cytokines for ~6 weeks, termed herein “continuous stimulation”. In parallel, TNBC cells were exposed to “short stimulation” of 48 h by TNFα + IL-1β. The images of Figure 1A indicate that short stimulation by the cytokines did not induce modifications in cell morphology, in both cell types; in contrast, the continuous stimulation by TNFα + IL-1β has changed TNBC cell morphology. In both BT-549 cells and MDA-MB-231 cells, following persistent cytokine stimulation cells with a flattened morphology could be detected; in parallel, cells with extended cellular protrusions were noted in BT-549 cells, but not in MDA-MB-231 cells.

To provide a quantitative indication to changes in cell morphology following continuous TNFα + IL-1β stimulation, IN Cell analyses were performed on TNBC cells following persistent cytokine/vehicle treatment. Analyses performed with calcein and Hoechst fluorescent staining have demonstrated definite alterations in morphology in both BT-549 and MDA-MB-231 cells following continuous TNFα + IL-1β stimulation (Figure 1B), which were quantitatively identified by significantly increased cell and nuclear areas after continuous cytokine stimulation (Figure 1B).

### 3.2. Continuous Stimulation by Proinflammatory Cytokines Modifies Gene Expression in TNBC Cells

**Figure 2:**
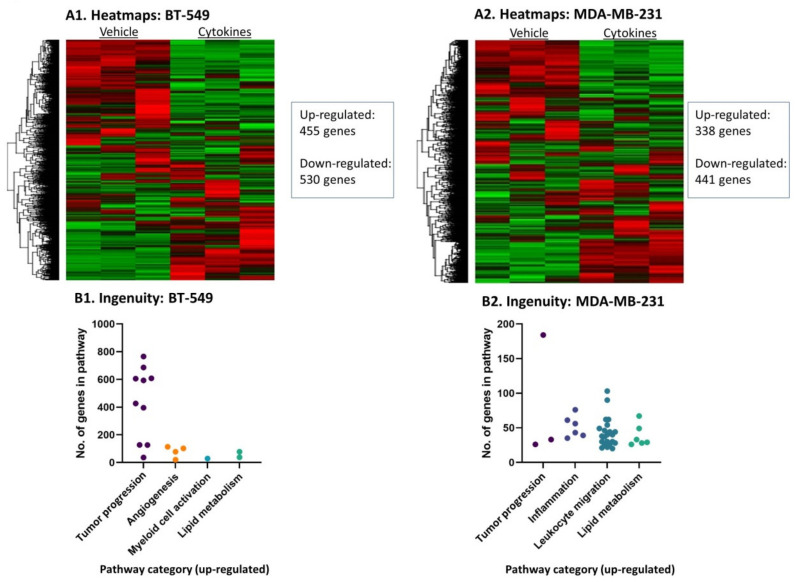
**Continuous TNFα + IL-1β stimulation leads to changes in transcriptional programs in TNBC cells.** BT-549 and MDA-MB-231 cells that were continuously stimulated by TNFα + IL-1β, or treated by a vehicle control (as described in Figure 1) were subjected to RNAseq analysis. (**A**) Heatmaps of all genes in (**A1**) BT-549 and (**A2**) MDA-MB-231 cells. (**B**) Differentially expressed genes that passed the cutoff FC ≥ 2 or FC ≤ −2 with pFDR < 0.05 were analyzed in Ingenuity program for pathway enrichment analyses. Significantly upregulated (Z-score > 2) annotations that were classified in cancer-related categories are presented in (**B1**) BT-549 cells and (**B2**) MDA-MB-231 cells. Each dot represents a category, whose detailed annotations and the number of genes in each annotation are demonstrated in Table S2 (BT-549 cells) and Table S3 (MDA-MB-231 cells).

To further investigate the impact of persistent stimulation by proinflammatory factors that are chronically present at the TME such as TNFα + IL-1β [13,14,18], TNBC cells that have undergone continuous treatment by the cytokines/vehicle were subjected to RNAseq analysis. The findings of Figure 2 indicate that following the persistent stimulation by TNFα + IL-1β, the expression of hundreds of genes was changed in both TNBC cell types. ANOVA statistical analysis, using cutoff of pFDR < 0.05 and fold change FC ≥ 2 or FC ≤ −2 between cytokine-stimulated cells and their vehicle-treated controls, revealed that the expression of 985 genes was modified in BT-549 cells (455 genes were upregulated and 530 were downregulated) (Figure 2A1) and 779 genes were differentially expressed in MDA-MB-231 cells (338 genes were upregulated and 441 were downregulated) (Figure 2A2).

Ingenuity pathway analyses of “Diseases and Functions” that were performed on the transcriptome data revealed similarities but also differences between the two TNBC cell types, following continuous stimulation by TNFα + IL-1β. The findings presented in Figure 2B demonstrate major cancer-related categories of ingenuity-defined upregulated annotations (activation Z score > 2) that were identified in each cell type; Tables S2 and S3 demonstrate the specific annotations included in each category and the number of genes included in each annotation. Overall, the cancer-related annotations included genes that are involved in general in tumor progression, angiogenesis, myeloid cell activation, inflammation, leukocyte migration and/or lipid metabolism, depending on the cell type. The two cell types shared upregulation in annotations that include genes generally involved in tumor progression and lipid metabolism (Figure 2B, Tables S2 and S3). Annotations typical of “inflammation” and “leukocyte migration” were enriched in MDA-MB-231 cells, demonstrating that these cells were particularly skewed into a proinflammatory phenotype (Figure 2B and Table S3).

**Figure 3:**
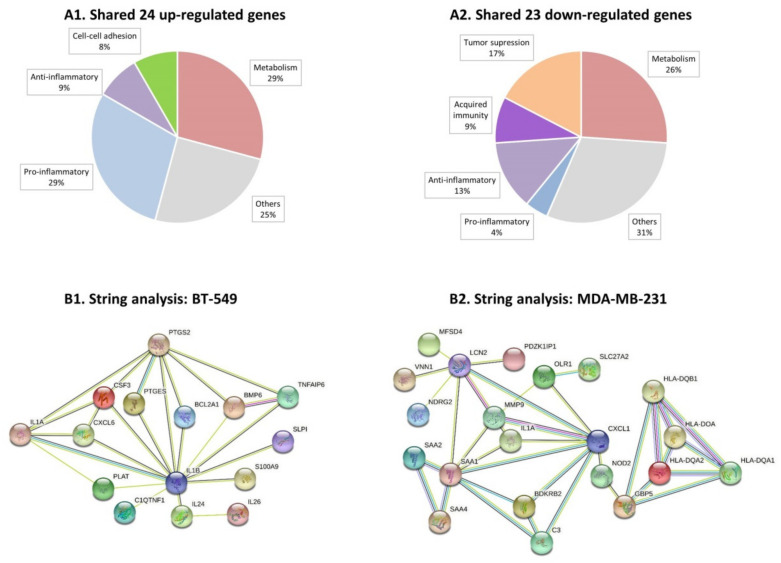
**Continuous TNFα + IL-1β stimulation skews TNBC cells towards a proinflammatory phenotype.** BT-549 and MDA-MB-231 cells that were continuously stimulated by TNFα + IL-1β, or treated by a vehicle control (as described in Figure 1) were subjected to RNAseq analysis (as described in Figure 2). (**A**) This part of the figure describes the categories to which differentially expressed genes—shared between BT-549 and MDA-MB-231 cells—may belong (differentially expressed: between cytokine-stimulated and vehicle control cells). (**A1**) 24 shared upregulated genes. (**A2**) 23 shared downregulated genes. The shared genes are indicated in Table S4A (shared upregulated genes) and Table S4B (shared downregulated genes). (**B**) Protein-protein association network analysis (STRING). Top 30 upregulated genes that passed the cutoff FC ≥ 2 or FC ≤ −2 with pFDR < 0.05 (Table S5) were analyzed in STRING. Color codes are provided herein: https://string-db.org/cgi/input?sessionId=bpw2uCSzWy2E&input_page_active_form=multiple_identifiers [31]. (**B1**) BT-549 cells. (**B2**) MDA-MB-231 cells. The names of 30 top upregulated genes are provided in Table S5 (Table S5A: BT-549 cells; Table S5B: MDA-Mb-231 cells). The names of 30 top downregulated genes are provided in Table S6 (Table S6A: BT-549 cells; Table S6B: MDA-MB-231 cells).

We then analyzed the characteristics of the genes that were modified by persistent TNFα + IL-1β stimulation in both TNBC cell types alike, namely: 24 protein-coding genes that were upregulated (pFDR < 0.05, FC ≥ 2) in both BT-549 and MDA-MB-231 cells (Table S4A), and 23 protein-coding genes that were downregulated (pFDR < 0.05, FC ≤ −2) in these two cell types (Table S4B). When we categorized these shared genes based on currently published information, we found that the shared upregulated genes were enriched by genes coding for proteins that are proinflammatory in nature, as well as by metabolism-related genes (Figure 3A1). The shared downregulated genes included mainly tumor-suppressor genes and genes coding for proteins that are involved in acquired immunity and inhibition of inflammation, as well as metabolism-related genes (Figure 3A2).

Then, we performed protein–protein association network analysis (STRING) on the top 30 upregulated genes in each of the TNBC cell types (Table S5A,B for BT-549 and MDA-MB-231 cells, respectively). In cytokine-stimulated BT-549 cells we noted associations between 15 of the proteins, with 11 of these proteins known as promoting inflammatory processes (Figure 3B1 and Table S5A). The proinflammatory genes that stood in the core of the network in BT-549 cells were CXCL6 and IL-1β itself, associated with other typical proinflammatory mediators such as IL-24, IL-26, CSF-3 and others (Figure 3B1). In cytokine-stimulated MDA-MB-231 cells, the STRING analysis also provided evidence to an inflammatory network: out of the 30 top upregulated genes, 21 genes were involved in a protein–protein network and 13 of the genes could be associated with inflammatory processes (Figure 3B2 and Table S5B). Here, the proinflammatory chemokine CXCL1 and SAA1 were at the center of the network, with other mediators that regulate inflammation directly or indirectly also being involved, such as OLR1, C3 and others. Of note, similar analysis of the 30 top downregulated genes in the two cell types (Table S6A,B) did not reveal any particular connections or shared characteristics of the genes.

Thus, in the two TNBC cell types, continuous TNFα + IL-1β stimulation has led to elevation in proinflammatory mediators that are connected to each other, with different proinflammatory networks established in BT-549 and MDA-MB-231 cells. A particularly strong proinflammatory phenotype was gained by MDA-MB-231 cells following persistent TNFα + IL-1β stimulation, as indicated by the Ingenuity analysis (Figure 2B2).

### 3.3. Continuous Stimulation by Proinflammatory Cytokines Promotes TNBC Cell Dispersion but Does Not Have a Consistent Impact on Stemness, Chemoresistance and Tumor Cell Proliferation

To determine if persistent TNFα + IL-1β stimulation modifies invasion-related functions in TNBC cells, we analyzed the ability of the cancer cells to undergo epithelial-to-mesenchymal transition (EMT). Gene expression studies (included in the RNAseq analyses) did not reveal a consistent pattern of upregulated expression of EMT-related genes such as snail, slug, zeb1 and zeb2 in BT-549 or MDA-MB-231 cells (data not shown). Moreover, analyses of vimentin and E-cadherin at the protein level did not provide evidence to EMT-related alterations in the cells (Figure S1); of note, in line with the fact that TNBC cells are mesenchymal in nature, vehicle-treated BT-549 and MDA-MB-231 cells did not express E-cadherin at all (Figure S1), therefore E-cadherin could also not be downregulated by the persistent cytokine stimulation.

**Figure 4:**
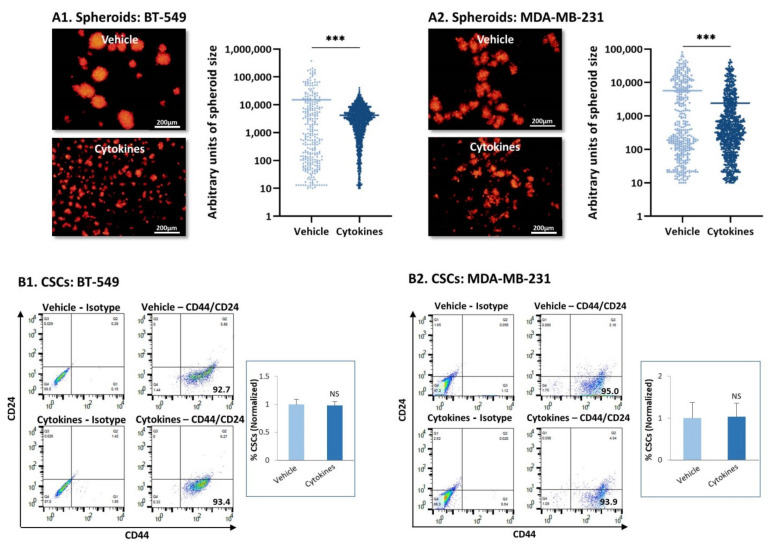
**Continuous TNFα + IL-1β stimulation leads to tumor cell dispersion and does not affect stemness in TNBC cells.** (**A**) mCherry-expressing TNBC cells were continuously stimulated by TNFα + IL-1β, or treated by a vehicle control (as described in Figure 1), and then have been tested for formation of tumor spheroids. (**A1**) BT-549 cells. (**A2**) MDA-MB-231 cells. The photos demonstrate the representative patterns of spheroid formation by the cells, at 3 days or 7 days after seeding, for BT-549 cells and MDA-MB-231 cells, respectively. Bar, 200 µm. The results of a representative experiment out of *n* = 3 are presented. Spheroid size was quantified by the ImageJ program in 10 fields for each treatment, in each experiment. *** *p* < 0.001. (**B**) Incidence of CSCs, determined as CD44+/CD24^low/^-cells. (**B1**) BT-549 cells. (**B2**) MDA-MB-231 cells. The figure presents flow cytometry plots of a representative experiment, and the graphs demonstrate the average ± SD of normalized values of CD44+/CD24^low/^-cells in *n* = 3 experiments; the levels of CSCs in vehicle-treated cells that were identified in the different experiments were given the value of 1. NS, not significant.

In line with the formation of cellular protrusions by BT-549 upon persistent stimulation by TNFα + IL-1β (Figure 1A1), these cells demonstrated elevated migratory potential in response to serum proteins (Figure S2A); MDA-MB-231 cells did not acquire higher migratory functions following continuous cytokine stimulation (Figure S2B). However, both cell types demonstrated high dispersion abilities upon persistent cytokine stimulation (Figure 4A). Following continuous TNFα + IL-1β stimulation, the two TNBC cell types formed fewer cell-to-cell contacts, remained dissociated from each other and generated only small tumor cell aggregates (Figure 4A). The difference between the cytokine-stimulated cells and the vehicle-treated cells was evident by the quantitative analyses, whose findings (in a logarithmic scale) are demonstrated in Figure 4A.

As tumor spheroids are formed primarily by cancer stem cells (CSCs) [32,33] and require the formation of cell-to-cell contacts, we next determined the stemness of TNBC cells that have been persistently stimulated by TNFα + IL-1β. The major characteristic of CSCs in breast cancer is that they express high CD44 levels and low/none CD24 levels and thus are identified as CD44^high^/CD24^low/^-cells [32,33]. As expected from their TNBC phenotype, more than 90% of the BT-549 and MDA-MB-231 cell population consisted of CSCs (Figure 4B); continuous TNFα + IL-1β stimulation had no effect on the proportion of the CSC sub-population in either of the TNBC cells (Figure 4B).

Taken together with the spheroid studies, these data indicate that stemness was not disrupted in TNBC exposed to persistent TNFα + IL-1β stimulation; rather, they revealed the preference of persistently cytokine-stimulated TNBC cells to remain scattered and dispersed. This phenotype may testify for increased spreading ability and metastatic potential.

**Figure 5:**
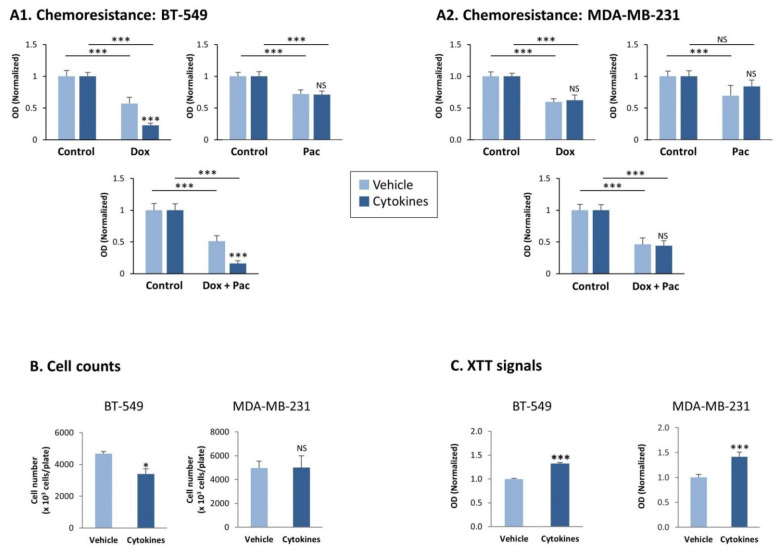
Continuous TNFα + IL-1β stimulation does not consistently affect chemoresistance and proliferation of TNBC cells, but leads to increased levels of mitochondrial enzymes in the tumor cells. BT-549 and MDA-MB-231 cells were continuously stimulated by TNFα + IL-1β, or treated by a vehicle control (as described in Figure 1). (**A**) Chemoresistance of BT-549 cells (**A1**) and of MDA-MB-231 cells (**A2**). The cells were grown for 24 h in the presence of the cytokines/vehicle, with doxorubicin (dox; 5 μM) and/or paclitaxel (pac; 350 nM). Control cells were grown in the presence of the solubilizer of the drugs. The results of a representative experiment of *n* > 3 are presented, where the OD values of the vehicle control group were given the value of 1. *** *p* < 0.001. NS, Not significant. (**B**) Cell counts, determining the numbers of BT-549 cells and MDA-MB-231 cells 72 h after seeding in similar numbers (cytokine- vs. vehicle-treated cells). Average ± SD values of *n* = 3 is presented for each cell type.* *p* < 0.05. NS, not significant. (**C**) XTT signals of BT-549 cells and MDA-MB-231 cells grown for 72 h after seeding in similar numbers (cytokine- vs. vehicle-treated cells). The XTT values of a representative experiment of *n* = 3 are presented, where the values in the vehicle control group were given the value of 1. *** *p* < 0.001. NS, not significant.

Because CSCs stand in the basis of chemoresistance [34], we also determined whether the consistent stimulation of TNBC cells by the cytokines has altered their sensitivity to chemotherapies that are routinely administered to TNBC patients, namely doxorubicin and paclitaxel. In these studies, we determined tumor cell sensitivity to each drug alone and to both of them together (Figure 5A). The data of Figure 5A indicate that each of the two chemotherapies downregulated tumor cell survival in both BT-549 and MDA-MB-231 cells. Both TNBC cell types did not demonstrate modified resistance to paclitaxel upon persistent cytokine stimulation; also, cytokine-stimulated MDA-MB-231 cells were as sensitive to doxorubicin and to the combination of both drugs together as did the vehicle-treated cells. The exception was identified in BT-549 cells, were we noticed reduced resistance to doxorubicin following consistent TNFα + IL-1β stimulation, which was standing in the basis of their increased sensitivity to the combined treatment by doxorubicin + paclitaxel together (Figure 5A).

Then, we determined the effects of continuous TNFα + IL-1β stimulation on tumor cell growth. Cell count studies revealed that continuous cytokine stimulation has downregulated cancer cell proliferation in BT-549 cells and did not affect tumor cell growth in MDA-MB-231 cells (Figure 5B). In contrast, XTT-based assays, which are conventionally used to determine cell viability, demonstrated that in both TNBC cell types the XTT signals were significantly higher in the persistently cytokine-stimulated cells than in their control vehicle-treated cells (Figure 5C).

### 3.4. Continuous Stimulation by Proinflammatory Cytokines Leads to Increased Glycolysis and OXPHOS in TNBC Cells

Unlike the cell count-based analyses that we have demonstrated in Figure 5B, the results of the XTT assays of Figure 5C detect the level of active mitochondrial enzymes in the cells. The significant increase in XTT signals in both BT-549 and MDA-MB-231 cells upon continuous stimulation by TNFα + IL-1β (Figure 5C) suggested that cytokine stimulation has modified the metabolic properties of the cells. This possibility was supported by the Ingenuity results of Figure 2B and the analyses of shared genes demonstrated in Figure 3, indicating that metabolism-related pathways were affected by the persistent stimulation of TNBC cell types by TNFα + IL-1β.

**Figure 6:**
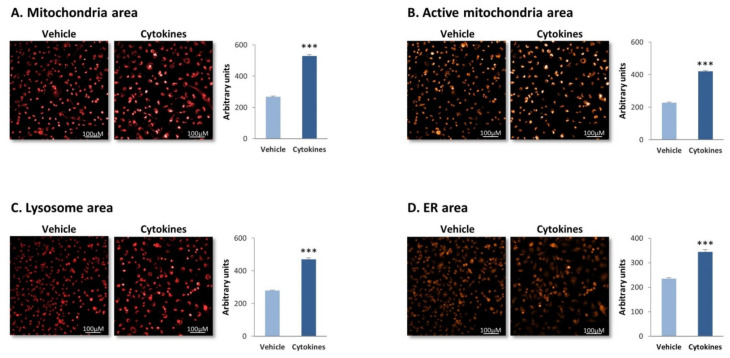
**Continuous TNFα + IL-1β stimulation increases the area of metabolism-associated organelles in TNBC cells.** BT-549 cells were continuously stimulated by TNFα + IL-1β, or treated by a vehicle control (as described in Figure 1), and the organization and alterations in metabolism-associated organelles were determined. (**A**) Mitochondria area, determined by MitoTracker-Deep Red staining. (**B**) Active mitochondria area, determined by TMRE staining. (**C**) Lysosome area, determined by lysotracker staining. (**D**) ER area, determined by ER-Hunt staining. Photos of cell morphology are demonstrated, as well as quantification of cell characteristics by the IN Cell technology. Bar, 100 μM. The results of a representative experiment of *n* = 3 are presented. *** *p* < 0.001.

To determine this possibility, we evaluated in BT-549 cells the status of cellular organelles that take part in cell metabolism using the IN Cell technology (MDA-MB-231 cells have detached from the designated IN Cell plates and therefore could not be studied in this type of analysis). Staining overall mitochondria by MitoTracker-Deep Red and active mitochondria by TMRE has revealed about twofold increase in mitochondria area and activity (Figure 6A,B, respectively). Moreover, a general elevation in the metabolic activity of the cells was reflected by elevated areas of the ER (ER-hunt staining) and of the lysosome (lysotracker staining) (Figure 6C,D).

**Figure 7:**
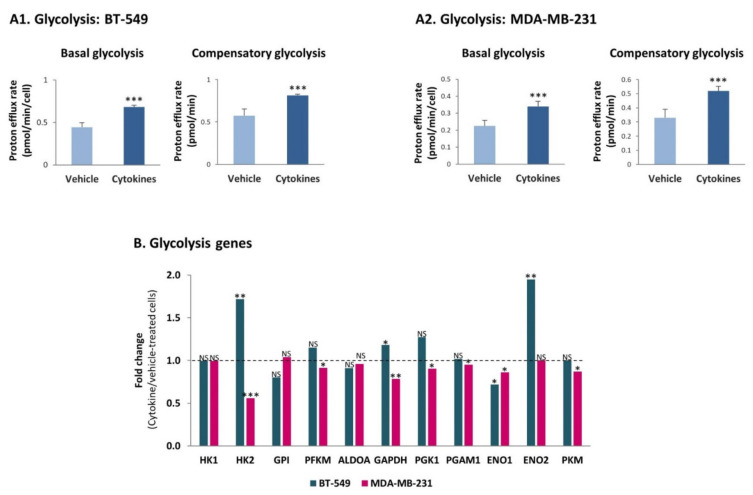
Continuous TNFα + IL-1β stimulation increases the glycolytic activity of TNBC cells, in a transcription independent manner. (**A**) Glycolytic activity. BT-549 and MDA-MB-231 cells were continuously stimulated by TNFα + IL-1β, or treated by a vehicle control (as described in Figure 1), and the glycolytic activities were determined by the seahorse technology. (**A1**) BT-549 cells. (**A2**) MDA-MB-231 cells. The results of a representative experiment of *n* = 3 are presented. *** *p* < 0.001. (**B**) Fold change in expression of genes coding for glycolysis enzymes, between cytokine-stimulated and vehicle-treated cells, determined by the transcriptome analyses (described in Figure 2). *** *p* < 0.001, ** *p* < 0.01, * *p* < 0.05. NS, Not significant.

These findings were followed by determining the ability of BT-549 and MDA-MB-231 cells to exert glycolysis and OXPHOS, the two major metabolic pathways that provide energy sources to the cells [35,36]. Using the seahorse technology, we found that persistent stimulation of TNBC cells with TNFα + IL-1β augmented the glycolytic ability of the two TNBC cells, increasing basal glycolysis as well as compensatory glycolysis (Figure 7A) which reflects the ability of the cells to produce energy under stress conditions. Our transcriptome analyses of the BT-549 and MDA-MB-231 cells indicated that the increase in the glycolytic activity was not accompanied by elevated transcription of key enzymes that take part in glycolysis (Figure 7B; PKM stands for PKM1 and PKM2).

**Figure 8:**
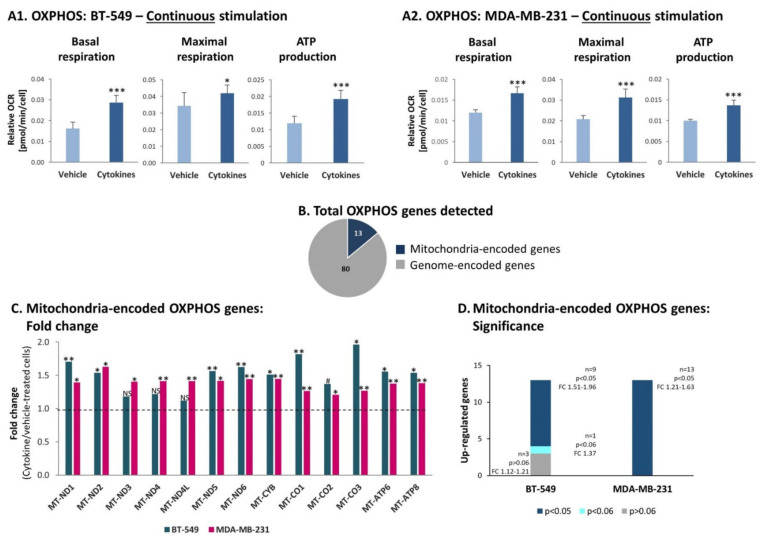
Continuous TNFα + IL-1β stimulation increases OXPHOS in TNBC cells, accompanied by elevated transcription levels of mitochondria-encoded OXPHOS genes. BT-549 and MDA-MB-231 cells were continuously stimulated by TNFα + IL-1β, or treated by vehicle control (as described in Figure 1). (**A**) OXPHOS-related activities were determined by the seahorse technology. (**A1**) BT-549 cells. (**A2**) MDA-MB-231 cells. The results of a representative experiment of n>3 are presented. *** *p* < 0.001, * *p* < 0.05. (**B**) A chart demonstrating the numbers of OXPHOS genes encoded in the nucleus genome and in mitochondria genome, identified in the transcriptome analyses of BT-549 cells and MDA-MB-231 cells (described in Figure 2). (**C**) Fold change in expression of mitochondria-encoded OXPHOS genes, comparing between cytokine-stimulated and vehicle-treated cells, determined by the transcriptome analyses (described in Figure 2). ** *p* < 0.01, * *p* < 0.05, # *p* < 0.06. NS, not significant. (**D**) A graph demonstrating the significance values of alterations observed in mitochondria-encoded OXPHOS genes in BT-549 cells and MDA-MB-231 cells. The alterations in gene expression were determined by the transcriptome analyses (described in Figure 2).

In parallel, OXPHOS levels were also increased upon continuous stimulation of BT-549 and MDA-MB-231 cells by TNFα + IL-1β. Under these conditions, significant elevations were noted in basal respiration rates, maximal respiration rates and ATP production in both TNBC cell types (Figure 8A). A similar analysis performed for both cell types under short-term conditions of TNFα + IL-1β stimulation did not reveal alterations in OXPHOS activities: basal respiration rates, maximal respiration rates and ATP production (Figure S3).

Unlike the glycolysis process, the increased OXPHOS functions noted upon persistent cytokine stimulation were accompanied by upregulation in transcription of mitochondria-encoded OXPHOS genes in both TNBC cell types. Out of 97 genes that participate in OXPHOS activities, we detected in the transcriptome analysis the expression of 93 genes in TNBC cells. Eighty of these genes are encoded in the nucleus genome (Figure 8B) and in general their expression was not affected by continuous cytokine stimulation, in both BT-549 and MDA-MB-231 cells (data not shown). However, all 13 mitochondria-encoded OXPHOS genes were upregulated in both cell types (Figure 8C); in BT-549 cells, nine out of these 13 genes were significantly elevated by continuous cytokine stimulation, and in MDA-MB-231 the elevation was significant in all 13 genes (Figure 8D).

Together, these findings indicate that continuous TNFα + IL-1β stimulation increased the glycolytic activities of TNBC cells in a non-transcription-mediated manner, and in parallel it elevated the expression of mitochondria-encoded OXPHOS genes and OXPHOS activity levels in these cells.

### 3.5. Continuous Stimulation by Proinflammatory Cytokines Leads via Metabolic Transition to Increased Activation of p65 in TNBC Cells

To determine which advantages may be given to TNBC cells by cytokine-increased glycolysis and OXPHOS, we measured lactate levels at the extracellular milieu of the cells and the levels of ATP in the cells. The findings of Figure S4 demonstrate that neither of these two energetic products were elevated in cytokine-stimulated TNBC cells, suggesting that the products generated by elevated levels of glycolysis and OXPHOS were exploited by the cancer cells at other levels.

**Figure 9:**
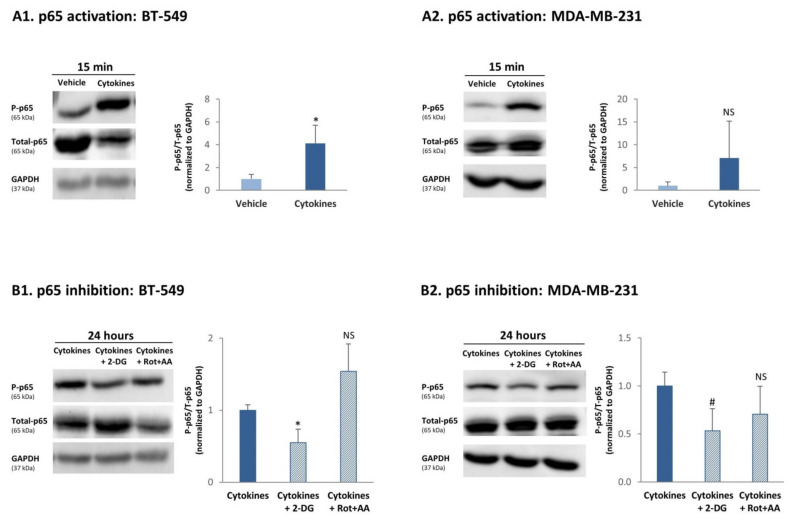
**Continuous TNFα + IL-1β stimulation induces a glycolysis-dependent process of p65 activation in TNBC cells.** (**A**) p65 activation. BT-549 and MDA-MB-231 cells were continuously stimulated by TNFα + IL-1β, or treated by vehicle control (as described in Figure 1). After being continuously treated by cytokines/vehicle, BT-549 cells (**A1**) and MDA-MB-231 cells (**A2**) were washed and treated by TNFα + IL-1β/vehicle for 15 min. The levels of p65 phosphorylation were determined in Western blot analyses as P-p65/Total-p65; each of the two signals was normalized to GAPDH (loading control) beforehand. The results of a representative experiment of *n* ≥ 3 are presented in the blots. The averaged results of *n* ≥ 3 are presented in the graphs (±SD), where the values of vehicle-treated cells in the different experiments were given the value of 1. * *p* < 0.05. NS, Not significant. The arbitrary phosphorylation values obtained in the three MDA-MB-231 experiments demonstrated in Table S7A indicate that a consistent upregulation of p65 phosphorylation was obtained following cytokine stimulation. (**B**) The roles of glycolysis and OXPHOS in inducing p65 activation. BT-549 and MDA-MB-231 TNBC cells were continuously stimulated by TNFα + IL-1β (as described in Figure 1). Following continuous cytokine stimulation, BT-549 cells (**B1**) and MDA-MB-231 cells (**B2**) were washed and stimulated by TNFα + IL-1β for 24 h (in order to enable the inhibitors to act) in the presence of a glycolysis inhibitor (2-DG) and OXPHOS inhibitors (Rot+AA), or their solubilizer (as described in the materials and methods section). The levels of p65 phosphorylation were determined in Western blot analyses as P-p65/Total-p65; each of the two signals was normalized to GAPDH (loading control) beforehand. The results of a representative experiment of *n* ≥ 3 are presented in the blots. The averaged results of *n* ≥ 3 are presented in the graphs (±SD), where the values of cytokine-treated cells—without inhibitors—in the different experiments were averaged and were given the value of 1. * *p* < 0.05, # *p* = 0.0505, NS = not significant. The arbitrary phosphorylation values obtained in the three MDA-MB-231 experiments demonstrated in Table S7B indicate that a consistent downregulation of p65 phosphorylation was obtained following 2-DG treatment.

To determine this possibility, we asked if increased cytokine-induced glycolysis and OXPHOS levels could promote the ability of TNBC cells to exploit better their TME. The results of Ingenuity pathway analysis (Figure 2B) proposed that inflammatory pathways may be highly relevant in this context. Because of the involvement of cancer inflammation in promoting tumor aggressiveness [3,4], we determined the impact of glycolysis and OXPHOS on the activation of p65, which is a canonical transcription factor mediating the proinflammatory activities of TNFα and IL-1β [37,38]. The findings of Figure 9 indicate that although BT-549 and MDA-MB-231 cells were continuously stimulated by TNFα + IL-1β for ~6 weeks, they were not refractory to this inflammatory stimulation and at the end of the persistent stimulatory process responded to 15-min TNFα + IL-1β stimulation (a typical time point in which p65 activation is noted) by increased p65 phosphorylation (accompanied by reduced expression of Total-p65; Figure 9A). Here, the averaged results of all experiments demonstrated significant elevations in p65 activation in cytokine-stimulated BT-549 cells, but not in cytokine-stimulated MDA-MB-231 cells. Of note, in MDA-MB-231 cells, upregulation of p65 activation due to persistent cytokine stimulation was consistent in three different independent experiments (Table S7A); it was the variance in the levels of basal phosphorylation and in the extent of upregulation of p65 activation between the different experiments, which has led to nonsignificant statistical analysis of the Western blot densitometry performed to all experiments together (Table S7A).

To determine whether glycolysis or OXPHOS had the ability to regulate p65 activation upon continuous TNFα + IL-1β stimulation, the cells were persistently stimulated by TNFα + IL-1β and at the last 24 h of growth they were cultured with 2-DG (glycolysis inhibitor) or Rot+AA (OXPHOS inhibitors); this time point was selected in order to enable the inhibitors to act. These studies indicated that 24 h following incubation with the glycolysis inhibitor, p65 activation by TNFα + IL-1β was reduced in both TNBC cells (Figure 9B); here again, downregulation of p65 by the glycolysis inhibitor (2-DG) in MDA-MB-231 cells was consistent in three different independent experiments (*p* = 0.0505; Table S7B); however, they came out only close-to-significant when considered all together (Figure 9B2) because of variability that was noted between the different experiments in the levels of phosphorylation in cells without inhibitors, and in the extent of decrease in p65 induced by 2-DG (Table S7B). In contrast to the effects of glycolysis on p65 activation, OXPHOS inhibitors did not alter the level of p65 activation in both cell types (Figure 9B and Table S7B).

Overall, our findings indicate that continuous TNFα + IL-1β stimulation has led through upregulation of glycolysis to induction of p65 activation in TNBC cells.

### 3.6. Continuous Stimulation of TNBC Cells by Proinflammatory Cytokines Leads in a Glycolysis-Dependent Manner to Increased Release of Proinflammatory Mediators and to Monocyte and Neutrophil Migration In Vivo

**Figure 10:**
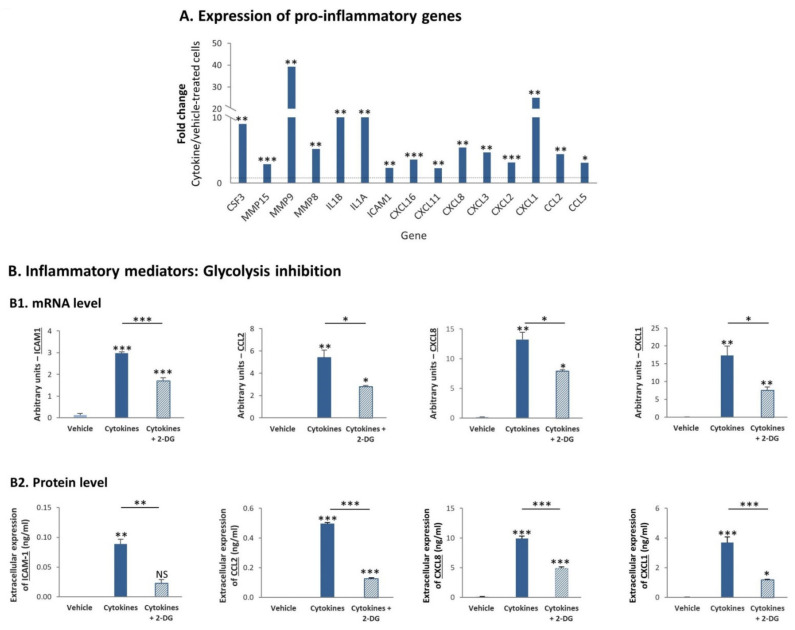
Continuous TNFα + IL-1β stimulation upregulates the mRNA and protein expression levels of proinflammatory mediators in TNBC cells, in a glycolysis-dependent process (**A**). MDA-MB-231 cells were continuously stimulated by TNFα + IL-1β, or treated by vehicle control (as described in Figure 1). Fold change in expression of proinflammatory genes between cytokine-stimulated and vehicle-treated cells was determined by the transcriptome analyses described in Figure 2. *** *p* < 0.001, ** *p* < 0.01, * *p* < 0.05. (**B**) mRNA levels and protein levels of proinflammatory mediators. (**B1**) mRNA levels were determined by quantitative RT-PCR. The results of a representative experiment of *n* = 3 are presented. *** *p* < 0.001, ** *p* < 0.01, * *p* < 0.05. (**B2**) Protein levels were determined by ELISA and are presented relative to cell numbers. The results of a representative experiment of *n* = 3 are presented. *** *p* < 0.001, ** *p* < 0.01, * *p* < 0.05. NS, Not significant.

In previous parts of the study we demonstrated by Ingenuity analysis that of the two TNBC cell types, MDA-MB-231 cells were particularly enriched with proinflammatory characteristics, following their persistent stimulation by TNFα + IL-1β (Figure 2B2). Within this proinflammatory phenotype of MDA-MB-231 cells that was indicated by the transcriptome analyses, we noticed significant elevation in the mRNA levels of key proinflammatory mediators—many of which are connected to tumor progression—following the continuous procedure of cytokine stimulation of MDA-MB-231 cells by TNFα + IL-1β (Figure 10A).

To follow up on the involvement of glycolysis in promoting the activation of p65 (Figure 9B2), which is a cardinal transcription factor involved in the regulation of many proinflammatory mediators [37,38], we determined if glycolysis regulates the transcription and protein expression of specific proinflammatory mediators in MDA-MB-231 cells that were persistently stimulated by TNFα + IL-1β. To address this question, we focused on: (1) soluble ICAM-1 (sICAM-1), whose elevated levels were connected to higher tumor aggressiveness in breast cancer patients [39,40,41]; (2) CCL2, the main inducer of monocyte migration to tumors, which is strongly connected to higher tumorigenicity in a large variety of cancers, breast cancer included [42,43,44,45,46,47,48]; (3) CXCL8 and CXCL1, two key inducers of neutrophil migration that are involved in regulating tumor progression [43,46,47,49,50].

We observed that the expression of ICAM-1, CCL2, CXCL8 and CXCL1 was increased at the mRNA level by continuous TNFα + IL-1β stimulation (Figure 10B1), agreeing with the results obtained in transcriptome analyses (Figure 10A). Moreover, the increased transcription of these proinflammatory mediators has led to their elevated expression at the protein level (Figure 10B2). In line with the fact that glycolysis elevated p65 activation, the transcription and protein expression levels of all of these factors were dependent on glycolysis (Figure 10B). However, OXPHOS—which did not regulate p65 activation in MDA-MB-231 cells (Figure 9B2)—controlled the protein expression of only two of the proinflammatory mediators—CXCL1 and CCL2 (Figure S5); moreover, OXPHOS did not regulate the mRNA levels of the four tested proinflammatory factors in a consistent manner, and its impact on protein levels did coincide with its effects on mRNA levels (data not shown).

**Figure 11:**
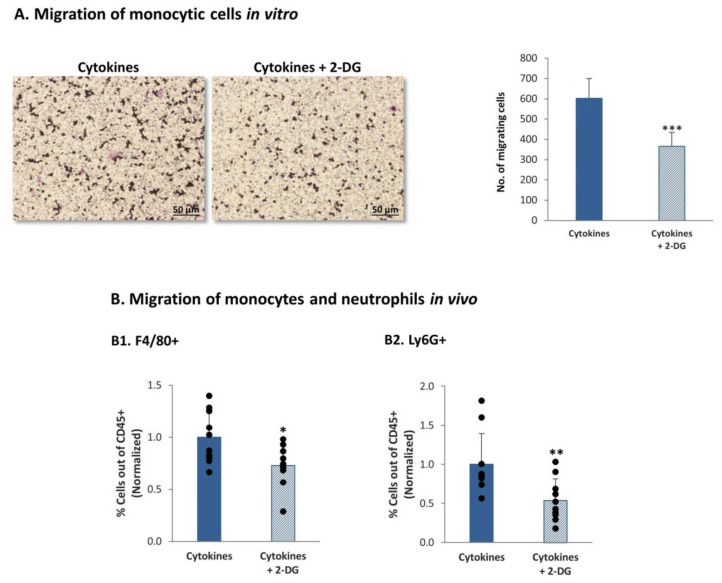
Continuous TNFα + IL-1β stimulation of TNBC cells leads to a glycolysis-dependent migration of monocytes and neutrophils in vivo (**A**) Migration of monocytic THP-1 cells in response to CM of MDA-MB-231 cells undergoing persistent stimulation by TNFα + IL-1β. MDA-MB-231 cells that were continuously stimulated by TNFα + IL-1β (as described in Figure 1) were treated with the inhibitor of glycolysis (2-DG) or its solubilizer. CM free of exogenously-added cytokines or 2-DG were collected (as described in materials and methods section) and were used in transwell migration assays of THP-1 cells. The figure demonstrates representative images of the migrating cells and quantitative analyses of migrating cells, in a representative experiment of *n* > 3. *** *p* < 0.001. Bar, 50 µm. (**B**) Migration of monocytes and neutrophils in vivo. MDA-MB-231 cells that were continuously stimulated by TNFα + IL-1β (as described in Figure 1) were treated with the inhibitor of glycolysis (2-DG) or its solubilizer, as described in the materials and methods section. CM free of exogenously-added cytokines or 2-DG were collected (as described in materials and methods section), filtered and injected with matrigel to form plugs in female Balb/C mice; Plugs were removed 7 days later, and infiltrating cells were recovered from the plugs and analyzed by flow cytometry. The percentage of F4/80+ cells (monocytes) (**B1**) and of Ly6G+ cells (neutrophils) (**B2**) out of CD45+ cells was determined. The results are of 10 mice in each group, obtained in two independent experimental repeats. Average ± SD of normalized values in *n* = 10 (in each group) are presented; the percentage of cells in plugs containing CM of cytokine-treated cells, without 2-DG, were averaged and were given the value of 1. ** *p* < 0.01, * *p* < 0.05.

In view of the consistent roles of glycolysis in regulating the expression of inflammatory chemokines, which are major regulators of leukocyte migration to tumors, we asked if glycolysis controls the migration of monocytic THP-1 cells. In these assays, we obtained CM from MDA-MB-231 cells that were persistently stimulated by TNFα + IL-1β in the absence or presence of the glycolysis inhibitor 2-DG or its solubilizer. The CM that were used in the migration assay did not include exogenously-added cytokines or 2-DG (please see more details in the “Materials and methods” section). These CM were then used in transwell experiments testing the migration of monocytic THP-1 cells. The results in Figure 11A demonstrate that glycolysis inhibition reduced the ability of the cytokine-deprived CM to induce the migration of the monocytic cells, indicating that metabolic processes can regulate the levels of factors that control leukocyte migration.

To determine the impact of glycolysis inhibition of leukocyte migration in the in vivo setting, matrigel plugs were generated; the plugs contained CM of TNFα + IL-1β-persistently stimulated MDA-MB-231 cells that were grown in the presence of the glycolysis inhibitor 2-DG or its solubilizer. Here again, the CM that were used in vivo did not contain exogenously-added cytokines or 2-DG (please see more details in the materials and methods section). The plugs were injected to the mammary fat pad of female mice and were removed 7 days later; then, the levels of monocytes and neutrophils were determined in the CD45+ leukocyte cell population. By using the F4/80 and Ly6G markers (identifying monocytes and neutrophils, respectively) we found that inhibition of glycolysis has significantly reduced the recruitment of monocytes and neutrophils to the plugs (Figure 11B).

Together, our observations provide novel evidence to the ability of chronic inflammation (represented by continuous TNFα + IL-1β stimulation) to promote the levels of glycolysis in TNBC cells; glycolysis, in turn, controlled the transcription and protein expression levels of factors regulating leukocyte migration, as indicated by reduced recruitment of monocytes and neutrophils in vivo.

## 4. Discussion

In the course of chronic tumor inflammation, cancer cells are continuously exposed to proinflammatory mediators that promote their prometastatic properties. In breast cancer, the proinflammatory and prometastatic cytokines TNFα and IL-1β are present in tumors together, from the time of malignant transformation and on, throughout the process of cancer progression [13,14,18]. Yet, the impact of persistent proinflammatory stimulation on breast tumor cells was not investigated in depth, leading us to determine the effects of continuous TNFα + IL-1β stimulation on TNBC cell characteristics and functions.

At first, our findings indicated that persistent TNFα + IL-1β stimulation of TNBC cells, but not short-term stimulation, has given rise to morphological changes in the cells, including the appearance of flat cells. The nature of these cells is not clear at this point, and could reflect senescent cells or alternatively polyaneuploid cells that have been recently connected to increased malignancy phenotype [51,52]. In either case, the entire cell population that was obtained following the consistent cytokine stimulation has demonstrated changes in gene expression and upregulated metabolic properties; the metabolic alterations included elevations in glycolysis and OXPHOS, and were mechanistically connected to increased transcription and production of proinflammatory factors by the cancer cells.

The research of metabolism in cancer has been much intensified during the last several years, but the interest in this topic goes back as early as the early years of the 20th century. At that time, Otto Warburg and his colleagues found that in the presence of oxygen, tumor cells exert increased glycolytic activities and proposed that mitochondrial respiration is defective in cancer cells [35,36,53]. This well-known “Warburg effect” has set the ground for the investigation of glycolysis and OXPHOS in cancer; with time, it was found that in many tumor types the mitochondria are not defective but rather that they are functional and that their activities are important for tumor establishment and metastasis [35,36].

The findings of our current study illustrate the ability of TNBC cells to exert elevated glycolytic functions alongside with increased OXPHOS activities, following their continuous stimulation by proinflammatory cytokines. In this case, the tumor cells demonstrate an important level of plasticity that enables them to respond to exogenous inflammatory stimuli by undergoing metabolic transition in both directions, towards increased levels of glycolysis as well as OXPHOS. By exerting elevated levels of both processes, the tumor cells can gain many advantages: increased glycolytic activities can provide the cancer cells with higher amounts of byproducts that are necessary for tumor cell growth, and in parallel increased OXPHOS levels can give the tumor cells high levels of flexibility in using a variable spectra of energy sources: not only glucose but also fatty acids and glutamine.

Thus, our observations suggest that chronic inflammation enables TNBC cells to gain the advantage of a hybrid metabolic phenotype that combines the benefits of both glycolysis and OXPHOS [54]. This hybrid state has been recently connected to a more aggressive tumor phenotype by several studies [54]. Most importantly, the research by Dupuy et al. demonstrated that highly metastatic murine breast tumor cells expressed increased glycolytic and OXPHOS activities than breast tumor cells with a lower metastatic potential [55]. In the current study, the heterogeneous nature of the TNBC cells—which was envisioned by different morphological states but may also take place at other levels following persistent TNFα + IL-1β stimulation—raises the possibility that some of the cells gained higher glycolytic activities while others attained increased OXPHOS activities. This way, different cell types may provide complementing advantages to the entire cancer cell population, eventually promoting prometastatic characteristics as a whole.

Our findings indicate that the advantages gained by the tumor cells are not at the levels of increased presence of extracellular lactate or intracellular ATP that could have been used as a source of energy. Here, it is possible that lactate and ATP have been already exploited by the cancer cells to support other needs, such as communication with other cells (as demonstrated for extracellular lactate [56]) or exerting biochemical functions that depend on transcription and protein synthesis of proinflammatory and tumor-promoting mediators, which were increased by the persistent proinflammatory stimulation.

In parallel, our data suggest that when TNBC cells acquired an elevated hybrid metabolic state as a result of chronic tumor-related inflammation, they can express higher flexibility in facing external stresses and in adapting to their microenvironment. Indeed, our study has provided novel evidence on the ability of glycolysis to upregulate the transcription (possibly through p65 activation) and consequently the protein expression levels of prometastatic inflammatory mediators such as sICAM-1 and chemokines in TNBC cells. These observations are in line with a recent study of hepatocellular carcinoma (HCC), demonstrating that glycolysis upregulated in a p65-dependent manner the expression levels of CXCL8 and CXCL2 in monocytes that were treated with tumor cell-derived supernatants [57]. Thus, our observations suggest that a metabolism-nuclear connection may have a major role in potentiating TNBC aggressiveness and add to recent findings proposing mechanistic connections between key metabolic pathways, gene regulation and protein activation [36,54,58,59].

Such processes of metabolism-regulated gene expression may control the prometastatic phenotype of the TME by increasing the presence of deleterious leukocyte subsets in the tumors. We demonstrated that glycolysis has upregulated the transcription of CCL2, CXCL8 and CXCL1, chemokines that were causatively connected to increased infiltration of monocytes and neutrophils to tumors. Accordingly, inhibition of glycolysis has led to significant reduction in monocyte and neutrophil migration in vivo. As these two leukocyte subsets are strongly connected to increased tumor progression, our findings propose novel roles for chronic inflammation in regulating via metabolic plasticity the recruitment of deleterious leukocyte subsets to TNBC tumors. This possibility is supported by the HCC study mentioned above, demonstrating that the glycolysis-induced chemokines played key roles in promoting neutrophil migration to tumors and that neutrophil presence was connected to elevated metastasis in patients [57]. Moreover, similar players, including CCL2 and CXCL8 were found by Sainz and his colleagues to be upregulated when pancreatic ductal adenocarcinoma cells have been persistently exposed for 14 days to galactose; this process has shifted the cells towards increased OXPHOS activities and was connected to immune-evasion [60].

Therefore, our study strongly connects between chronic inflammation and regulation of metabolism in TNBC, suggesting that such a connection may lead to deleterious impacts in terms of tumor progression. We demonstrated that in response to continuous proinflammatory stimulation, in this case by TNFα and IL-1β, TNBC cells have been skewed strongly in the direction of proinflammatory phenotype that was connected with metabolic plasticity. Our research agrees well with other research works that demonstrated the impact of inflammation-metabolism interaction on tumor progression [57,61,62]. Specifically in TNBC, our data suggest that a vicious cycle takes place in TNBC tumors through metabolic transition in the cancer cells: this cycle is driven by chronic inflammation, leading through metabolic plasticity in the tumor cells to exacerbated levels of prometastatic chemokines that can upregulate the recruitment of deleterious leukocyte subsets and chronic inflammation.

In addition to TME-mediated effects, the chemokines may also regulate the intrinsic prometastatic activities of the cancer cells, as suggested by recent studies in breast cancer, demonstrating that the prometastatic chemokine CCL5 promoted the metabolic activity of the cancer cells; stimulation of the cancer cells by this chemokine has led to increased glycolysis-dependent tumor growth and invasion in vitro and to elevated ability to form tumors in vivo [61,62]. Although CCL5 was not regulated in our system of persistent TNFα + IL-1β stimulation of TNBC cells, it is highly possible that chemokines such as CCL2, CXCL8 and CXCL1—which were increased due to the continuous cytokine stimulation and are known as having multiple promalignancy activities that are exerted on the cancer cells [43,63]—may upregulate tumor-promoting activities in the tumor cells themselves. Through such an activity, a complete cycle of positive feedbacks can be achieved, in which inflammation-driven increase in metabolism gives rise to elevated levels of prometastatic chemokines that then act back on the TME and on the cancer cells, to eventually upregulate their malignancy potential.

Overall, we believe that our observations may have important clinical implications because they set the hybrid phenotype of increased glycolysis + OXPHOS activities as a much better target for therapy than targeting only one of the metabolic processes alone. The connection of a hybrid metabolic state to elevated tumor aggressiveness suggests that cancer cells may be deprived more efficiently from their energetic and metabolite sources if both glycolysis and OXPHOS will be reduced. Moreover, the inflammation-metabolism connection that we have witnessed in TNBC cells proposes that the influence of metabolic inhibitors targeting both glycolysis and OXPHOS may be enhanced if they will be joined by anti-inflammatory drugs. In this respect, nonsteroidal anti-inflammatory drugs (NSAID) such as aspirin may apply, in view of their strong connection to reduced malignancy risk in variety of malignant diseases, including breast cancer [64,65].

## 5. Conclusions

Our study has exemplified the complexity of cancer-TME interactions by demonstrating that continuous stimulation of TNBC cells by proinflammatory cytokines that are constantly expressed in breast tumors—such as TNFα + IL-1β—lead to a hybrid metabolic state that is characterized by elevated glycolysis as well as OXPHOS, and skew TNBC into a proinflammatory phenotype that is regulated by glycolysis in the cancer cells. This increase in metabolic pathways, which is gained by TNBC cells upon continuous proinflammatory stimulation, may serve their needs in multiple manners that expand from providing higher amounts of metabolites, through increased production of prometastatic chemokines, to elevated levels of deleterious leukocytes at the TME. These findings may have clinical importance in setting up combination therapies that may improve the prognosis of TNBC patients.

## Data Availability

https://www.ncbi.nlm.nih.gov/geo/query/acc.cgi?acc=GSE169018. Accessed date is 16 March 2021.

## Supplementary Materials

The following are available online at https://www.mdpi.com/article/10.3390/cells10061356/s1, Figure S1: Expression of EMT markers by TNBC cells stimulated continuously by proinflammatory cytokines, Figure S2: Migration of TNBC cells that have been continuously stimulated by proinflammatory cytokines, Figure S3: Short-term TNFα + IL-1β stimulation does not increase OXPHOS in TNBC cells, Figure S4: Levels of extracellular lactate and ATP in TNBC cells continuously stimulated by proinflammatory cytokines, Figure S5: OXPHOS activities do not regulate the expression of inflammatory mediators that are induced by continuous cytokine stimulation in TNBC cells, Table S1: Primers used for quantitative RT-PCR analysis, Table S2: Continuous TNFα + IL-1β stimulation increases cancer-related annotations in BT-549 cells, Table S3: Continuous TNFα + IL-1β stimulation increases cancer-related annotations in MDA-MB-231 cells, Table S4: Genes that are regulated similarly by continuous TNFα + IL-1β in BT-549 and MDA-MB-231 TNBC cells, Table S5: Top 30 up-regulated genes in TNBC cells following continuous TNFα + IL-1β stimulation, Table S6: Top 30 down-regulated genes in TNBC cells following continuous TNFα + IL-1β stimulation, Table S7: Continuous TNFα + IL-1β stimulation induces a glycolysis-dependent process of p65 activation in MDA-MB-231 cells.

## References

[1] Zaidi M.R. (2019). The Interferon-Gamma Paradox in Cancer. J. Interf. Cytokine Res..

[2] Aqbi H.F., Wallace M., Sappal S., Payne K.K., Manjili M.H. (2018). IFN-gamma orchestrates tumor elimination, tumor dormancy, tumor escape, and progression. J. Leukoc Biol..

[3] Shalapour S., Karin M. (2015). Immunity, inflammation, and cancer: An eternal fight between good and evil. J. Clin. Investig..

[4] Colotta F., Allavena P., Sica A., Garlanda C., Mantovani A. (2009). Cancer-related inflammation, the seventh hallmark of cancer: Links to genetic instability. Carcinogenesis.

[5] Crusz S.M., Balkwill F.R. (2015). Inflammation and cancer: Advances and new agents. Nat. Rev. Clin. Oncol.

[6] Cruceriu D., Baldasici O., Balacescu O., Berindan-Neagoe I. (2020). The dual role of tumor necrosis factor-alpha (TNF-alpha) in breast cancer: Molecular insights and therapeutic approaches. Cell Oncol..

[7] Martinez-Reza I., Diaz L., Garcia-Becerra R. (2017). Preclinical and clinical aspects of TNF-alpha and its receptors TNFR1 and TNFR2 in breast cancer. J. Biomed. Sci..

[8] Al-Hatamleh M.A.I., Ahmad S., Boer J.C., Lim J., Chen X., Plebanski M., Mohamud R. (2019). A Perspective Review on the Role of Nanomedicine in the Modulation of TNF-TNFR2 Axis in Breast Cancer Immunotherapy. J. Oncol..

[9] Salamanna F., Borsari V., Contartese D., Costa V., Giavaresi G., Fini M. (2019). What Is the Role of Interleukins in Breast Cancer Bone Metastases? A Systematic Review of Preclinical and Clinical Evidence. Cancers.

[10] Tulotta C., Ottewell P. (2018). The role of IL-1B in breast cancer bone metastasis. Endocr. Relat. Cancer.

[11] Apte R.N., Voronov E. (2017). Immunotherapeutic approaches of IL-1 neutralization in the tumor microenvironment. J. Leukoc. Biol..

[12] Zhou X.L., Fan W., Yang G., Yu M.X. (2014). The clinical significance of PR, ER, NF- kappa B, and TNF- alpha in breast cancer. Dis. Mark..

[13] Soria G., Ofri-Shahak M., Haas I., Yaal-Hahoshen N., Leider-Trejo L., Leibovich-Rivkin T., Weitzenfeld P., Meshel T., Shabtai E., Gutman M. (2011). Inflammatory mediators in breast cancer: Coordinated expression of TNFalpha & IL-1beta with CCL2 & CCL5 and effects on epithelial-to-mesenchymal transition. BMC Cancer.

[14] Jin L., Yuan R.Q., Fuchs A., Yao Y., Joseph A., Schwall R., Schnitt S.J., Guida A., Hastings H.M., Andres J. (1997). Expression of interleukin-1beta in human breast carcinoma. Cancer.

[15] Semesiuk N.I., Zhylchuk A., Bezdenezhnykh N., Lykhova A., Vorontsova A.L., Zhylchuk V.E., Kudryavets Y.I. (2013). Disseminated tumor cells and enhanced level of some cytokines in bone marrow and peripheral blood of breast cancer patients as predictive factors of tumor progression. Exp. Oncol..

[16] Abrahamsson A., Morad V., Saarinen N.M., Dabrosin C. (2012). Estradiol, tamoxifen, and flaxseed alter IL-1beta and IL-1Ra levels in normal human breast tissue in vivo. J. Clin. Endocrinol. Metab..

[17] Perrier S., Caldefie-Chezet F., Vasson M.P. (2009). IL-1 family in breast cancer: Potential interplay with leptin and other adipocytokines. FEBS Lett..

[18] Chavey C., Bibeau F., Gourgou-Bourgade S., Burlinchon S., Boissiere F., Laune D., Roques S., Lazennec G. (2007). Oestrogen receptor negative breast cancers exhibit high cytokine content. Breast Cancer Res..

[19] Molnar I.A., Molnar B.A., Vizkeleti L., Fekete K., Tamas J., Deak P., Szundi C., Szekely B., Moldvay J., Vari-Kakas S. (2017). Breast carcinoma subtypes show different patterns of metastatic behavior. Virchows Arch..

[20] Abdelhakiem M.K., Johnstone C., Bergom C., Currey A., Robbins J.R. (2020). The influence of breast cancer subtype on survival after palliative radiation for osseous metastases. Cancer Med..

[21] Harbeck N., Penault-Llorca F., Cortes J., Gnant M., Houssami N., Poortmans P., Ruddy K., Tsang J., Cardoso F. (2019). Breast cancer. Nat. Rev. Dis. Primers.

[22] Cai X., Cao C., Li J., Chen F., Zhang S., Liu B., Zhang W., Zhang X., Ye L. (2017). Inflammatory factor TNF-alpha promotes the growth of breast cancer via the positive feedback loop of TNFR1/NF-kappaB (and/or p38)/p-STAT3/HBXIP/TNFR1. Oncotarget.

[23] Qiao Y., He H., Jonsson P., Sinha I., Zhao C., Dahlman-Wright K. (2016). AP-1 is a key regulator of proinflammatory cytokine TNFalpha-mediated triple-negative breast cancer progression. J. Biol. Chem..

[24] Kim S., Choi J.H., Kim J.B., Nam S.J., Yang J.H., Kim J.H., Lee J.E. (2008). Berberine suppresses TNF-alpha-induced MMP-9 and cell invasion through inhibition of AP-1 activity in MDA-MB-231 human breast cancer cells. Molecules.

[25] Liubomirski Y., Lerrer S., Meshel T., Rubinstein-Achiasaf L., Morein D., Wiemann S., Korner C., Ben-Baruch A. (2019). Tumor-Stroma-Inflammation Networks Promote Pro-metastatic Chemokines and Aggressiveness Characteristics in Triple-Negative Breast Cancer. Front. Immunol..

[26] Liubomirski Y., Lerrer S., Meshel T., Morein D., Rubinstein-Achiasaf L., Sprinzak D., Wiemann S., Korner C., Ehrlich M., Ben-Baruch A. (2019). Notch-Mediated Tumor-Stroma-Inflammation Networks Promote Invasive Properties and CXCL8 Expression in Triple-Negative Breast Cancer. Front. Immunol..

[27] Naldini A., Filippi I., Miglietta D., Moschetta M., Giavazzi R., Carraro F. (2010). Interleukin-1beta regulates the migratory potential of MDAMB231 breast cancer cells through the hypoxia-inducible factor-1alpha. Eur. J. Cancer.

[28] Chung S.T., Geerts D., Roseman K., Renaud A., Connelly L. (2017). Osteoprotegerin mediates tumor-promoting effects of Interleukin-1beta in breast cancer cells. Mol. Cancer.

[29] Dobin A., Davis C.A., Schlesinger F., Drenkow J., Zaleski C., Jha S., Batut P., Chaisson M., Gingeras T.R. (2013). STAR: Ultrafast universal RNA-seq aligner. Bioinformatics.

[30] Edgar R., Domrachev M., Lash A.E. (2002). Gene Expression Omnibus: NCBI gene expression and hybridization array data repository. Nucleic Acids Res..

[31] Szklarczyk D., Gable A.L., Lyon D., Junge A., Wyder S., Huerta-Cepas J., Simonovic M., Doncheva N.T., Morris J.H., Bork P. (2019). STRING v11: Protein-protein association networks with increased coverage, supporting functional discovery in genome-wide experimental datasets. Nucleic Acids Res..

[32] Brooks M.D., Burness M.L., Wicha M.S. (2015). Therapeutic Implications of Cellular Heterogeneity and Plasticity in Breast Cancer. Cell Stem Cell.

[33] Sanchez-Tillo E., Liu Y., de Barrios O., Siles L., Fanlo L., Cuatrecasas M., Darling D.S., Dean D.C., Castells A., Postigo A. (2012). EMT-activating transcription factors in cancer: Beyond EMT and tumor invasiveness. Cell Mol. Life Sci..

[34] De Angelis M.L., Francescangeli F., Zeuner A. (2019). Breast Cancer Stem Cells as Drivers of Tumor Chemoresistance, Dormancy and Relapse: New Challenges and Therapeutic Opportunities. Cancers.

[35] Zheng J. (2012). Energy metabolism of cancer: Glycolysis versus oxidative phosphorylation (Review). Oncol. Lett..

[36] Ward P.S., Thompson C.B. (2012). Metabolic reprogramming: A cancer hallmark even warburg did not anticipate. Cancer Cell.

[37] Zhang Q., Lenardo M.J., Baltimore D. (2017). 30 Years of NF-kappaB: A Blossoming of Relevance to Human Pathobiology. Cell.

[38] Kunnumakkara A.B., Shabnam B., Girisa S., Harsha C., Banik K., Devi T.B., Choudhury R., Sahu H., Parama D., Sailo B.L. (2020). Inflammation, NF-kappaB, and Chronic Diseases: How are They Linked?. Crit. Rev. Immunol..

[39] Bewick M., Conlon M., Lee H., Parissenti A.M., Zhang L., Gluck S., LaFrenie R.M. (2004). Evaluation of sICAM-1, sVCAM-1, and sE-Selectin levels in patients with metastatic breast cancer receiving high-dose chemotherapy. Stem Cells Dev..

[40] Thielemann A., Baszczuk A., Kopczynski Z., Nowak A., Grodecka-Gazdecka S. (2014). The clinical usefulness of assessing the concentration of cell adhesion molecules sVCAM-1 and sICAM-1 in the serum of women with primary breast cancer. Współczesna Onkologia.

[41] Touvier M., Fezeu L., Ahluwalia N., Julia C., Charnaux N., Sutton A., Mejean C., Latino-Martel P., Hercberg S., Galan P. (2013). Association between prediagnostic biomarkers of inflammation and endothelial function and cancer risk: A nested case-control study. Am. J. Epidemiol..

[42] Borsig L., Wolf M.J., Roblek M., Lorentzen A., Heikenwalder M. (2013). Inflammatory chemokines and metastasis-tracing the accessory. Oncogene.

[43] Morein D., Erlichman N., Ben-Baruch A. (2020). Beyond Cell Motility: The Expanding Roles of Chemokines and Their Receptors in Malignancy. Front. Immunol..

[44] Poeta V.M., Massara M., Capucetti A., Bonecchi R. (2019). Chemokines and Chemokine Receptors: New Targets for Cancer Immunotherapy. Front. Immunol..

[45] Do H.T.T., Lee C.H., Cho J. (2020). Chemokines and their Receptors: Multifaceted Roles in Cancer Progression and Potential Value as Cancer Prognostic Markers. Cancers.

[46] Del Prete A., Schioppa T., Tiberio L., Stabile H., Sozzani S. (2017). Leukocyte trafficking in tumor microenvironment. Curr. Opin. Pharmacol..

[47] Svensson S., Abrahamsson A., Rodriguez G.V., Olsson A.K., Jensen L., Cao Y., Dabrosin C. (2015). CCL2 and CCL5 Are Novel Therapeutic Targets for Estrogen-Dependent Breast Cancer. Clin. Cancer Res..

[48] Lim S.Y., Yuzhalin A.E., Gordon-Weeks A.N., Muschel R.J. (2016). Targeting the CCL2-CCR2 signaling axis in cancer metastasis. Oncotarget.

[49] Ha H., Debnath B., Neamati N. (2017). Role of the CXCL8-CXCR1/2 Axis in Cancer and Inflammatory Diseases. Theranostics.

[50] Acharyya S., Oskarsson T., Vanharanta S., Malladi S., Kim J., Morris P.G., Manova-Todorova K., Leversha M., Hogg N., Seshan V.E. (2012). A CXCL1 paracrine network links cancer chemoresistance and metastasis. Cell.

[51] Pienta K.J., Hammarlund E.U., Brown J.S., Amend S.R., Axelrod R.M. (2021). Cancer recurrence and lethality are enabled by enhanced survival and reversible cell cycle arrest of polyaneuploid cells. Proc. Natl. Acad. Sci. USA.

[52] Pienta K.J., Hammarlund E.U., Austin R.H., Axelrod R., Brown J.S., Amend S.R. (2020). Cancer cells employ an evolutionarily conserved polyploidization program to resist therapy. Seminars in Cancer Biology.

[53] Otto A.M. (2016). Warburg effect(s)-a biographical sketch of Otto Warburg and his impacts on tumor metabolism. Cancer Metab..

[54] Jia D., Park J.H., Jung K.H., Levine H., Kaipparettu B.A. (2018). Elucidating the Metabolic Plasticity of Cancer: Mitochondrial Reprogramming and Hybrid Metabolic States. Cells.

[55] Dupuy F., Tabaries S., Andrzejewski S., Dong Z., Blagih J., Annis M.G., Omeroglu A., Gao D., Leung S., Amir E. (2015). PDK1-Dependent Metabolic Reprogramming Dictates Metastatic Potential in Breast Cancer. Cell Metab..

[56] Naik A., Decock J. (2020). Lactate Metabolism and Immune Modulation in Breast Cancer: A Focused Review on Triple Negative Breast Tumors. Front. Oncol..

[57] Peng Z.P., Jiang Z.Z., Guo H.F., Zhou M.M., Huang Y.F., Ning W.R., Huang J.H., Zheng L., Wu Y. (2020). Glycolytic activation of monocytes regulates the accumulation and function of neutrophils in human hepatocellular carcinoma. J. Hepatol..

[58] Castegna A., McVicar D.W., Campanella A., Palmieri E.M., Menga A., Porporato P.E. (2020). Editorial: Metabolism Meets Function: Untangling the Cross-Talk Between Signaling and Metabolism. Front. Oncol..

[59] Smolkova K., Plecita-Hlavata L., Bellance N., Benard G., Rossignol R., Jezek P. (2011). Waves of gene regulation suppress and then restore oxidative phosphorylation in cancer cells. Int J. Biochem Cell Biol..

[60] Valle S., Alcala S., Martin-Hijano L., Cabezas-Sainz P., Navarro D., Munoz E.R., Yuste L., Tiwary K., Walter K., Ruiz-Canas L. (2020). Exploiting oxidative phosphorylation to promote the stem and immunoevasive properties of pancreatic cancer stem cells. Nat. Commun..

[61] Gao D., Cazares L.H., Fish E.N. (2017). CCL5-CCR5 interactions modulate metabolic events during tumor onset to promote tumorigenesis. BMC Cancer.

[62] Gao D., Rahbar R., Fish E.N. (2016). CCL5 activation of CCR5 regulates cell metabolism to enhance proliferation of breast cancer cells. Open Biol..

[63] Baram T., Rubinstein-Achiasaf L., Ben-Yaakov H., Ben-Baruch A. (2020). Inflammation-Driven Breast Tumor Cell Plasticity: Stemness/EMT, Therapy Resistance and Dormancy. Front. Oncol..

[64] Gaziano J.M. (2019). Aspirin for Primary Prevention: Clinical Considerations in 2019. JAMA.

[65] Kehm R.D., Hopper J.L., John E.M., Phillips K.A., MacInnis R.J., Dite G.S., Milne R.L., Liao Y., Zeinomar N., Knight J.A. (2019). Regular use of aspirin and other non-steroidal anti-inflammatory drugs and breast cancer risk for women at familial or genetic risk: A cohort study. Breast Cancer Res..

