# Supplementary material for: Continuous Inflammatory Stimulation Leads via Metabolic Plasticity to a Prometastatic Phenotype in Triple-Negative Breast Cancer Cells
Source: Cells. 2021 May 31;10(6):1356. doi: 10.3390/cells10061356 (PMC8229065; doi:10.3390/cells10061356)
Supplement: Supplementary file 1 [file cells-10-01356-s001.zip › cells-1183525-supplementary.pdf]

Figure S1

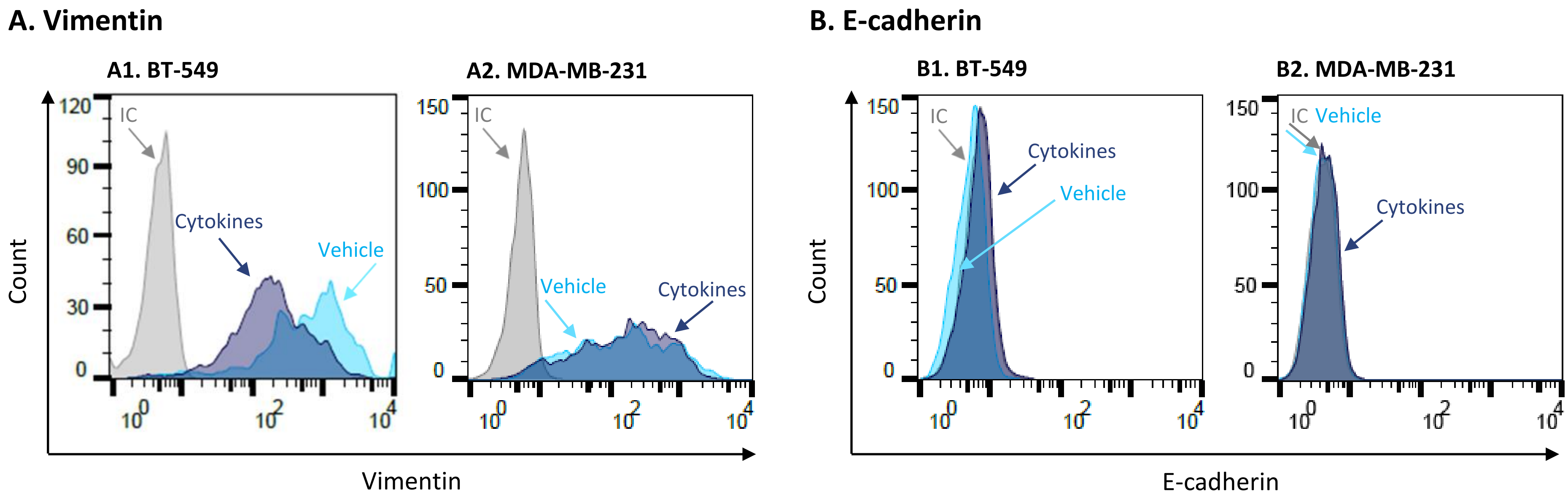

Expression of EMT markers by TNBC cells stimulated continuously by proinflammatory cytokines

BT-549 and MDA-MB-231 cells were continuously stimulated by TNFα + IL-1β, or treated by vehicle control (as described in Fig. 1). Expression of EMT markers was determined by flow cytometry. **(A)** Vimentin levels. Representative experiments of n≥3 for (A1) BT-549 cells and (A2) MDA-MB-231 cells are presented. **(B)** E-cadherin levels. Representative experiments of n≥3 for (B1) BT-549 and (B2) MDA-MB-231 cells are presented. IC, Non-relevant isotype control.

Figure S2

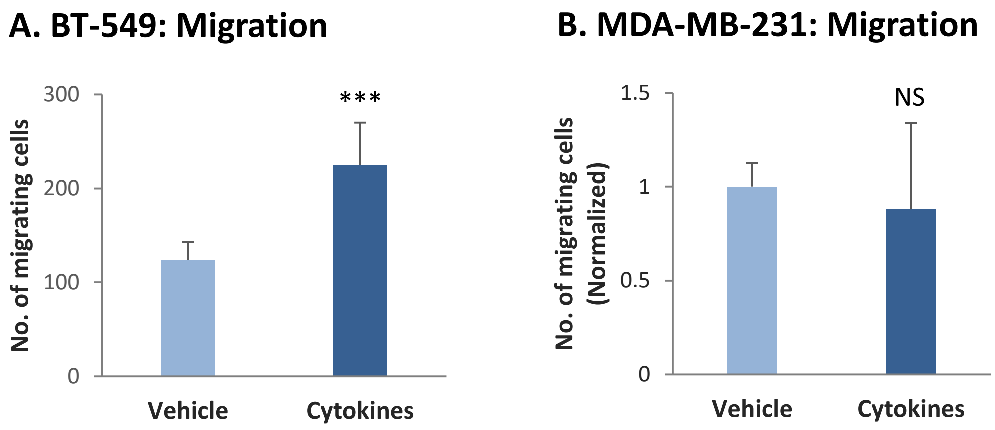

Migration of TNBC cells that have been continuously stimulated by proinflammatory cytokines

BT-549 **(A)** and MDA-MB-231 **(B)** cells were continuously stimulated by TNFα + IL-1β, or treated by vehicle control (as described in Fig. 1). Cell migration was determined in fibronectin-coated transwells in response to serum proteins. The migration of BT-549 cells is demonstrated as a representative experiment of n>3. The migration of MDA-MB-231 cells is demonstrated as the average of n=5 experiments, in which the value of vehicle-treated cells was given the value of 1. ***p<0.001, NS, Not significant.

Figure S3

A. OXPHOS: BT-549 – <u>Short</u> stimulation

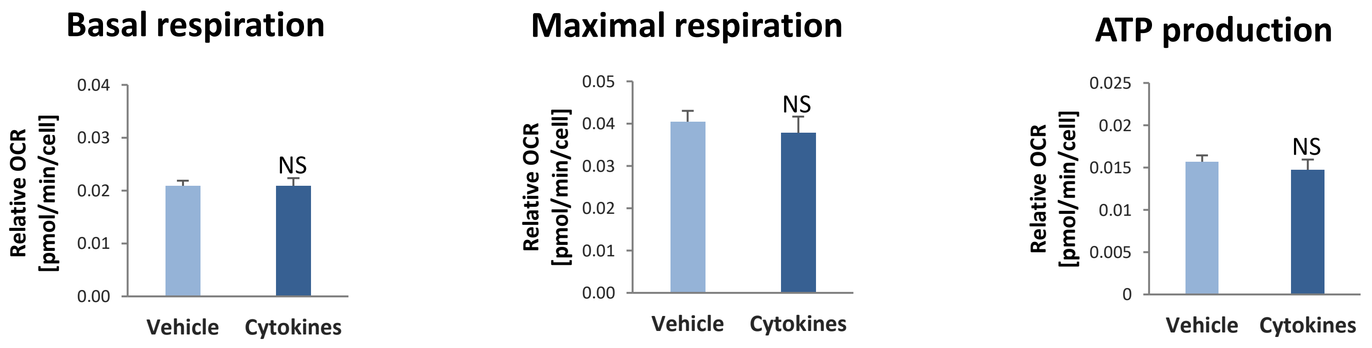

B. OXPHOS: MDA-MB-231 – <u>Short</u> stimulation

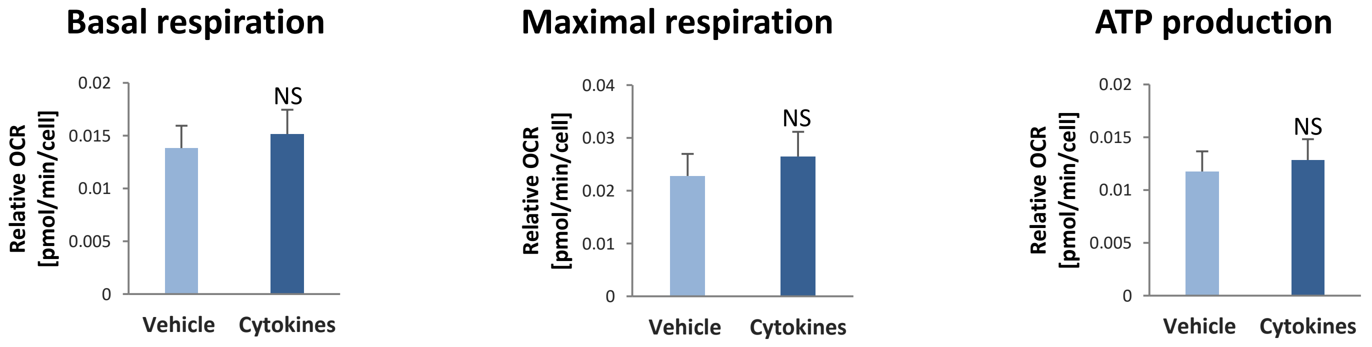

Short-term TNFα + IL-1β stimulation does not increase OXPHOS in TNBC cells

BT-549 and MDA-MB-231 cells were exposed to short-term stimulation (48 hours) by TNFα + IL-1β, or treated by vehicle control (as described in Fig. 1). OXPHOS activities were determined by the seahorse technology. The results of a representative experiment of n>3 are presented. NS, Not significant.

Figure S4

A. Extracellular lactate

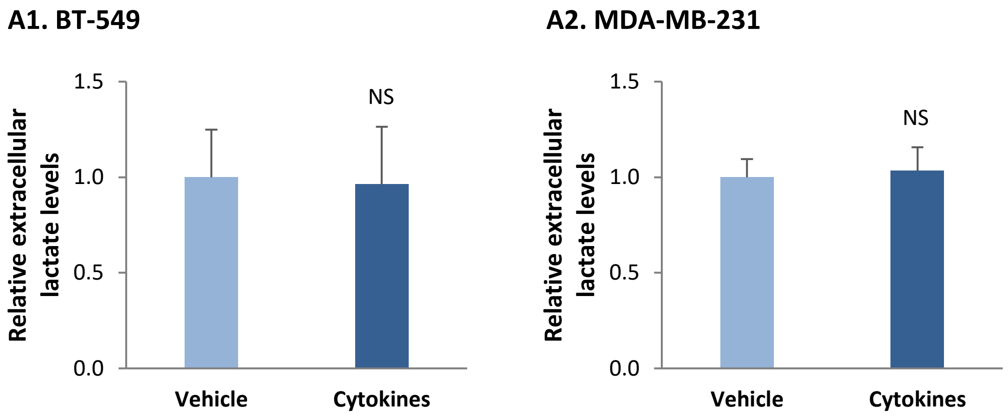

B. ATP

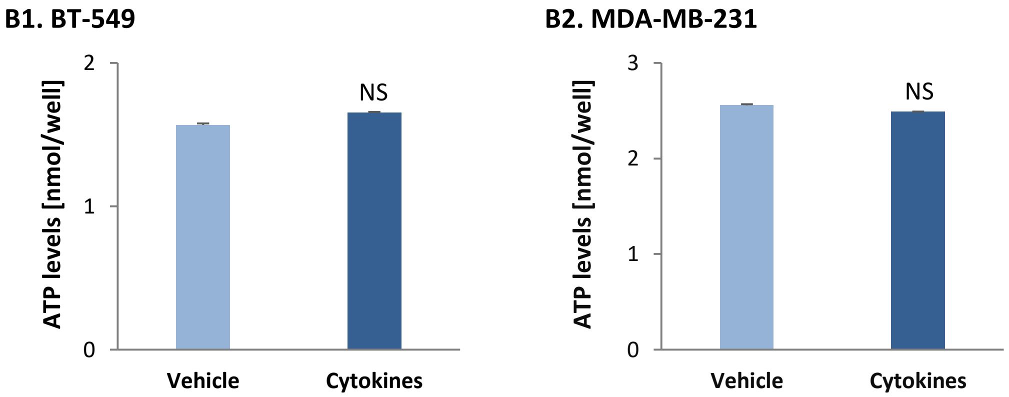

Levels of extracellular lactate and ATP in TNBC cells continuously stimulated by proinflammatory cytokines
BT-549 and MDA-MB-231 cells were continuously stimulated by TNFα + IL-1β, or treated by vehicle control (as described in Fig. 1). **(A)** Extracellular levels of lactate were measured in CM. The results are demonstrated as the average of n=3 experiments, in which the value of vehicle-treated cells was given the value of 1. **(B)** ATP levels were determined in cell lysates. The results of a representative experiment of n=3 are presented. NS, Not significant.

Figure S5

Inflammatory mediators: OXPHOS inhibition

Protein level

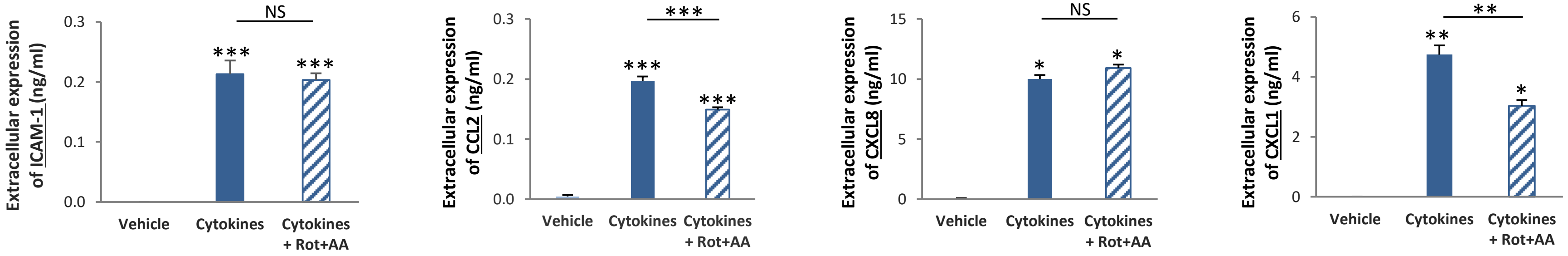

OXPHOS activities do not regulate the expression of inflammatory mediators that are induced by continuous cytokine stimulation in TNBC cells

MDA-MB-231 cells were continuously stimulated by TNFα + IL-1β, or treated by vehicle control (as described in Fig. 1). Protein levels of pro-inflammatory mediators were determined as described in “Materials and methods”; they were determined by ELISA and are presented relative to cell numbers. The results of a representative experiment of n=3 are presented. ***$p<0.001$, **$p<0.01$, *$p<0.05$. NS, Not significant.

Table S1

Gene	Sense primer (5'-3')	Anti-sense primer (5'-3')
ICAM1	GATGGGCAGTCAACAGCTAA	AGCGTAGGGTAAGGTTCTTG
CXCL8	TTCTGCAGCTCTGTGTGAAG	CAGTGTGGTCCACTCTCAAT
CXCL1	GCGCAGCAGGAGCGTCCGT	ATGCAGGATTGAGGCAAGCTTTC
CCL2	AGTCTCTGCCGCCCTTCT	GTGACTGGGGCATTGATTG
RS9	AACTTATGTGACCCCGCGGA	CAGCTTCAGCTCTTGGTCGA

Primers used for quantitative RT-PCR analysis.

Table S2

Category	"Diseases and Functions" annotations	p-value	Activation z-score	No. of genes in annotation
**Tumor progression**	Advanced malignant tumor	6.21E-16	2.01	126
	Advanced stage tumor	3.02E-16	2.01	127
	Cancer	2.54E-33	2.48	765
	Cancer of cells	3.33E-17	2.64	395
	Development of carcinoma	6.02E-23	2.25	593
	Development of malignant tumor	5.46E-26	2.43	605
	Formation of solid tumor	4.22E-32	2.28	686
	Frequency of tumor	2.1E-25	2.27	608
	Growth of solid tumor	3.48E-07	2.31	36
	Neoplasia of cells	1.77E-15	2.33	426
**Angiogenesis**	Development of vasculature	6.33E-13	3.11	114
	Vasculogenesis	3.54E-08	2.44	78
	Angiogenesis	1.31E-11	3.11	102
	Angiogenesis of lesion	2.79E-07	2.30	20
**Myeloid cell activation**	Activation of myeloid cells	7.01E-06	2.12	29
**Lipid metabolism**	Concentration of fatty acid	2.57E-08	2.25	38
	Concentration of lipid	1.82E-07	2.12	78

Continuous TNFα + IL-1β stimulation increases cancer-related annotations in BT-549 cells

BT-549 cells were continuously stimulated by TNFα + IL-1β, or treated by vehicle control (as described in Fig. 1). Transcriptome analysis was performed as described in Figure 2. Differentially expressed genes (cutoff: FC≥2 or FC≤-2, pFDR<0.05) were subjected to Ingenuity pathway analysis (as described in Figure 2). The Table presents significantly up-regulated annotations (z-score>2) divided into cancer-related categories.

Table S3

Category	"Diseases and Functions" annotations	p-value	Activation z-score	No. of genes in annotation
**Tumor progression**	Proliferation of tumor cells	7.61E-08	2.7	33
	Proliferation of cancer cells	0.00000082	2.4	26
	Mammary tumor	0.00000032	2.0	184
**Inflammation**	Interaction of leukocytes	5.47E-07	2.1	35
	Inflammatory response	3.28E-12	3.0	76
	Homing of leukocytes	1.4E-10	2.5	43
	Binding of blood cells	1.52E-07	2.1	39
	Activation of leukocytes	3.64E-09	3.6	56
	Activation of blood cells	3.69E-09	3.0	61
**Leukocyte migration**	Transmigration of leukocytes	3.19E-09	2.1	22
	Migration of phagocytes	1.47E-07	2.0	30
	Migration of mononuclear leukocytes	1.37E-08	2.1	40
	Leukocyte migration	4.8E-18	2.1	103
	Infiltration by neutrophils	1.92E-09	2.3	26
	Chemotaxis of phagocytes	5.71E-07	2.8	30
	Chemotaxis of neutrophils	0.00000049	2.6	20
	Chemotaxis of myeloid cells	3.04E-07	2.5	30
	Chemotaxis of leukocytes	8.79E-10	2.7	40
	Chemotaxis of granulocytes	0.00000169	2.2	21
	Cellular infiltration by phagocytes	2.9E-10	2.8	37
	Cellular infiltration by myeloid cells	1.42E-09	2.6	38
	Cellular infiltration by granulocytes	2.24E-08	2.1	28
	Cellular infiltration	2.51E-11	2.3	54
	Cell movement of T lymphocytes	0.00000181	2.1	26
	Cell movement of phagocytes	7.1E-12	2.7	62
	Cell movement of neutrophils	5.08E-13	2.7	44
	Cell movement of myeloid cells	4.95E-12	2.6	62
	Cell movement of mononuclear leukocytes	2E-09	2.4	49
	Cell movement of lymphatic system cells	4.33E-09	2.0	44
	Cell movement of leukocytes	3.83E-17	2.3	90
	Cell movement of granulocytes	3.04E-11	2.2	46
**Lipid metabolism**	Synthesis of lipid	4.97E-08	3.3	67
	Synthesis of fatty acid	6.76E-08	2.5	33
	Synthesis of eicosanoid	4.33E-08	2.9	26
	Metabolism of polyunsaturated fatty acids	3.68E-08	3.0	29
	Metabolism of eicosanoid	8.22E-08	3.0	28
	Fatty acid metabolism	4.88E-07	2.2	49

Continuous TNFα + IL-1β stimulation increases cancer-related annotations in MDA-MB-231 cells

MDA-MB-231 cells were continuously stimulated by TNFα + IL-1β, or treated by vehicle control (as described in Fig. 1). Transcriptome analysis was performed as described in Figure 2. Differentially expressed genes (cutoff: FC≥2 or FC≤-2, pFDR<0.05) were subjected to Ingenuity pathway analysis (as described in Figure 2). The Table presents significantly up-regulated annotations (z-score>2) divided into cancer-related categories.

Tables S4

A. Shared up-regulated genes

Gene	BT-549 fold change	MDA MB-231 fold change	BT-549 p-value	MDA-MB-231 p-value
OLR1	1021.3	43.6	0.002	0.001
IL24	434.2	11.3	0.000	0.001
C1QTNF1	101.9	7.0	0.004	0.005
IL1A	97.5	13.0	0.001	0.001
TEPP	41.2	2.7	0.000	0.002
CLDN3	30.8	2.2	0.005	0.001
TFPI2	30.0	2.6	0.004	0.000
MAP3K8	25.4	3.2	0.002	0.000
TNFAIP3	24.5	2.6	0.000	0.000
GAL	20.4	4.1	0.001	0.002
LRIG1	11.5	2.8	0.005	0.000
ASS1	10.8	2.7	0.003	0.002
IGSF3	7.7	4.1	0.000	0.000
CLDN1	5.1	5.4	0.002	0.004
ELOVL7	5.0	2.2	0.001	0.001
AKR1B1	4.3	3.1	0.001	0.000
CXCL1	4.1	25.1	0.004	0.005
SLC16A1	3.7	3.0	0.000	0.005
WWC1	3.4	3.6	0.001	0.000
MMP15	3.2	2.9	0.002	0.001
SLC22A23	3.1	3.5	0.004	0.000
MARCH3	2.9	2.6	0.003	0.004
VDR	2.8	2.2	0.001	0.004
ADAP2	2.7	5.1	0.002	0.000

B. Shared down-regulated genes

Gene	BT-549 fold change	MDA MB-231 fold change	BT-549 p-value	MDA-MB-231 p-value
APOBEC3G	-69.7	-2.6	0.003	0.004
RSAD2	-64.9	-2.5	0.003	0.001
CHRDL1	-44.3	-2.7	0.000	0.004
NDP	-38.0	-4.5	0.001	0.002
PLEKHG4B	-31.4	-34.2	0.003	0.001
IFIT2	-30.0	-2.0	0.002	0.005
OASL	-27.8	-2.6	0.002	0.003
SAMD9L	-15.4	-2.5	0.003	0.002
CMPK2	-15.0	-8.1	0.000	0.003
KAZALD1	-14.8	-2.1	0.000	0.003
PDE1C	-10.0	-4.4	0.001	0.001
ITGA10	-9.8	-9.6	0.000	0.000
EPHA5	-7.2	-6.7	0.000	0.000
HOPX	-5.0	-6.9	0.001	0.004
AIM1	-4.3	-2.6	0.000	0.000
GPSM3	-3.2	-2.0	0.001	0.002
TIMP3	-3.0	-2.8	0.002	0.001
PYGB	-2.9	-2.3	0.001	0.000
MAP2K6	-2.9	-2.6	0.002	0.001
SOCS3	-2.7	-2.1	0.000	0.003
SLC4A8	-2.6	-4.6	0.002	0.001
EHD3	-2.4	-2.4	0.000	0.005
AFF3	-2.4	-7.8	0.003	0.000

Genes that are regulated similarly by continuous TNFα + IL-1β in BT-549 and MDA-MB-231 TNBC cells

BT-549 and MDA-MB-231 cells were continuously stimulated by TNFα + IL-1β, or treated by vehicle control (as described in Fig. 1). Transcriptome analyses were performed as described in Figure 2. **(A)** Shared up-regulated genes (FC≥2, pFDR<0.05). **(B)** Shared down-regulated genes (FC≤-2, pFDR<0.05) are demonstrated.

Table S5

A. BT-549: 30 top up-regulated genes

Gene	Fold change	p-value
CSF3	1971.8	0.000
S100A9	1449.1	0.001
OLR1	1021.3	0.002
IL24	434.2	0.000
IL1B	290.2	0.001
TNFAIP6	215.9	0.002
SLPI	150.2	0.001
IL26	147.6	0.003
GCSAM	114.2	0.004
BCL2A1	112.2	0.000
C1QTNF1	101.9	0.004
IL1A	97.5	0.001
PTGS2	91.4	0.003
DNER	81.0	0.001
CYP39A1	71.4	0.004
PTGES	65.8	0.001
BMP6	64.5	0.003
TPBGL	54.1	0.003
TNS4	51.8	0.001
CXCL6	50.9	0.000
VNN3	49.9	0.000
SPOCD1	46.9	0.002
CACNA1A	45.0	0.002
PTPRD	41.5	0.001
TEPP	41.2	0.000
PLAT	37.5	0.002
DYSF	36.1	0.005
FEZ1	34.0	0.002
WNK4	33.4	0.001
CHI3L2	33.0	0.000

B. MDA-MB-231: 30 top up-regulated genes

Gene	Fold change	p-value
HLA-DQA1	291.5	0.003
NOD2	124.6	0.000
HLA-DQA2	112.2	0.000
LCN2	109.7	0.002
SLC27A2	100.6	0.000
PDZK1IP1	68.4	0.000
AZGP1	52.2	0.001
OLR1	43.6	0.001
WNT10A	40.8	0.000
MMP9	39.3	0.005
ALOX5AP	37.9	0.006
KCNN3	33.5	0.001
SAA4	30.3	0.001
C3	28.1	0.000
CXCL1	25.1	0.005
BDKRB2	24.3	0.003
VNN1	23.2	0.001
MFSD4A	22.2	0.003
HLA-DQB1	20.6	0.000
GBP5	20.5	0.004
NDRG2	18.8	0.000
CYP4X1	17.6	0.006
SAA2	17.0	0.000
SAA1	13.6	0.000
IL1A	13.0	0.001
C4orf26	12.7	0.000
TNIP3	11.9	0.001
HLA-DOA	11.7	0.000
CHST4	11.5	0.000
IL24	11.3	0.001

Top 30 up-regulated genes in TNBC cells following continuous TNFα + IL-1β stimulation
BT-549 and MDA-MB-231 cells were continuously stimulated by TNFα + IL-1β, or treated by vehicle control (as described in Fig. 1). Transcriptome analyses were performed as described in Figure 2. Tables of the top 30 up-regulated genes (cutoff: FC≥2, pFDR<0.05) are presented for **(A)** BT-549 and **(B)** MDA-MBA-231.

Table S6

A. BT-549: 30 top down-regulated genes

Gene	Fold change	p-value
LUZP2	-478.3	0.000
BST2	-340.5	0.001
SDPR	-206.9	0.003
IFITM1	-143.2	0.000
RGS4	-104.0	0.004
CHRM3	-104.0	0.003
FMN1	-78.4	0.001
MX1	-73.9	0.002
KCNK3	-69.9	0.000
APOBEC3G	-69.7	0.003
RSAD2	-64.9	0.003
DKK3	-52.9	0.002
LGALS3BP	-52.3	0.001
TM4SF18	-48.9	0.000
ELFN1	-48.2	0.001
IL15RA	-45.2	0.000
CHRDL1	-44.3	0.000
CDH11	-43.1	0.002
GIMAP2	-42.3	0.002
HNF4G	-39.4	0.000
NDP	-38.0	0.001
DEF6	-31.5	0.001
PLEKHG4B	-31.4	0.003
SH3TC2	-31.0	0.001
IFIT2	-30.0	0.002
PCDHGC5	-28.9	0.000
GBP3	-28.1	0.004
OASL	-27.8	0.002
IFI30	-26.5	0.000
CDK15	-26.4	0.001

B. MDA-MB-231: 30 top down-regulated genes

Gene	Fold change	p-value
HOXC5	-6160550.0	0.004
HSD3B1	-172.4	0.003
SLC30A10	-42.4	0.000
PLEKHG4B	-34.2	0.001
S100P	-31.8	0.000
ID4	-25.7	0.002
PCDH18	-21.9	0.000
MGMT	-21.7	0.002
GAS7	-21.3	0.000
TFF1	-19.9	0.000
SLC14A1	-19.8	0.002
NCALD	-18.4	0.002
KIF6	-18.1	0.005
RAB37	-18.0	0.001
C5orf46	-14.8	0.002
AGR2	-14.7	0.001
LAIR2	-14.5	0.000
PROX1	-14.2	0.000
FAM110B	-13.8	0.000
C9orf152	-13.7	0.001
PCDHB8	-13.7	0.005
RERG	-13.4	0.002
TSPAN8	-12.4	0.000
ADGRG2	-12.3	0.000
NPR3	-10.9	0.000
SHISA3	-10.8	0.001
BMP2	-10.3	0.002
ATP10A	-10.3	0.001
INHBB	-10.3	0.000
SOX4	-10.3	0.000

Top 30 down-regulated genes in TNBC cells following continuous TNFα + IL-1β stimulation

BT-549 and MDA-MB-231 cells were continuously stimulated by TNFα + IL-1β, or treated by vehicle control (as described in Fig. 1). Transcriptome analyses were performed as described in Figure 2. Tables of the top 30 down-regulated genes (cutoff: FC≤-2, pFDR<0.05) are presented for **(A)** BT-549 and **(B)** MDA-MBA-231.

Table S7

A. MDA-MB-231: 15 min of stimulation

	Arbitrary phosphorylation values (Normalized)	
	**Vehicle**	**Cytokines**
**Exp. 1**	1.96	16.39
**Exp. 2**	0.42	1.66
**Exp. 3**	0.62	3.15

B. MDA-MB-231: 24 hours of stimulation

	Arbitrary phosphorylation values (Normalized)		
	**Cytokines**	**Cytokines + 2-DG**	**Cytokines + Rot+AA**
**Exp. 1**	1.02	0.38	0.46
**Exp. 2**	0.85	0.42	0.64
**Exp. 3**	1.13	0.80	1.02

Continuous TNFα + IL-1β stimulation induces a glycolysis-dependent process of p65 activation in MDA-MB-231 cells
The Table complements the data demonstrated in Figure 9.
MDA-MB-231 cells were continuously stimulated by TNFα + IL-1β, or treated by vehicle control (as described in Fig. 1).
(**A**) p65 phosphorylation following 15 minutes of treatment by TNFα + IL-1β or vehicle. The Table demonstrates the normalized arbitrary phosphorylation values obtained by densitometry for each treatment in the different experiments, where the average of vehicle-treated cells was given the value of 1. **(B)** p65 phosphorylation following 24 hours of treatment by TNFα + IL-1β in the presence of glycolysis inhibitor (2-DG) or OXPHOS inhibitors (Rot+AA), or their solubilizer. The Table demonstrates the normalized arbitrary phosphorylation units obtained by densitometry for each treatment in the different experiments, where the average of the cytokines (without inhibitors)-stimulated cells was given the value of 1.
